# Biological effects of particulate matter samples during the COVID-19 pandemic: a comparison with the pre-lockdown period in Northwest Italy

**DOI:** 10.1007/s11869-023-01381-6

**Published:** 2023-06-06

**Authors:** Marta Gea, Manuela Macrì, Daniele Marangon, Francesco Antonio Pitasi, Marco Fontana, Tiziana Schilirò, Sara Bonetta

**Affiliations:** 1 Department of Public Health and Pediatrics, University of Torino, Via Santena 5 Bis, 10126 Turin, Italy; 2 Department of Life Sciences and Systems Biology, University of Torino, Via Accademia Albertina 13, 10123 Turin, Italy; 3 Regional Agency for Environmental Protection of Piedmont (ARPA Piemonte), Via Sabaudia 164, 10095 Grugliasco, Italy

**Keywords:** COVID-19 lockdown, Particulate matter, Cytotoxicity, Genotoxicity, Mutagenicity, Estrogenic activity

## Abstract

In 2020, during the COVID-19 pandemic, containment measures were applied inducing potential changes in air pollutant concentrations and thus in air toxicity. This study evaluates the role of restrictions on biological effects of particulate matter (PM) in different Northwest Italy sites: urban background, urban traffic, rural, and incinerator. Daily PM samples collected in 2020 were pooled according to restrictions: January/February (no restrictions), March and April (first lockdown), May/June and July/August/September (low restrictions), October/November/December (second lockdown). The 2019 samples (pre-pandemic period) were pooled as 2020 for comparison. Pools were extracted with organic solvents and extracts were tested to assess cytotoxicity (WST-1 assay) and genotoxicity (comet assay) on BEAS-2B cells, mutagenicity (Ames test) on TA98 and TA100 *Salmonella typhimurium* strains, and estrogenic activity (gene reporter assay) on MELN cells. Pollutant concentrations were also analyzed (PM_10_, PM_2.5_, polycyclic aromatic hydrocarbons). No difference was observed for PM and polycyclic aromatic hydrocarbon concentrations between 2020 and 2019. During lockdown months (2020), PM cytotoxicity/genotoxicity was significantly lower in some sites than during 2019, while considering PM mutagenicity/estrogenic activity some differences were detected but without statistical significance. PM extract effects decreased in some sites during 2020; this may be due to lockdowns that reduced/modified pollutant emissions and may be related also to complex PM origin/formation and to meteorological conditions. In conclusion, the study confirms that PM biological effects cannot be assessed considering only the PM concentration and suggests to include a battery of bioassay for air quality monitoring in order to protect human health from air pollution effects.

**Graphical Abstract:**

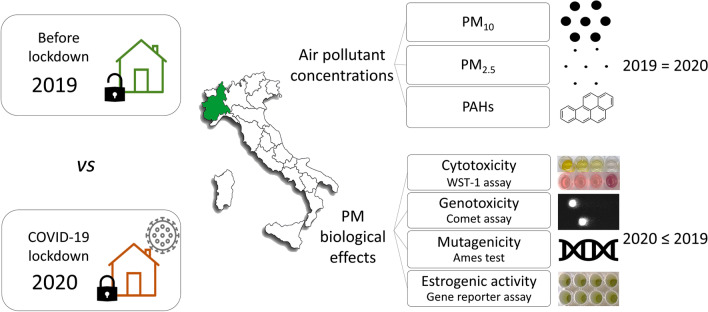

**Supplementary Information:**

The online version contains supplementary material available at 10.1007/s11869-023-01381-6.

## Introduction

On 11 March 2020, the World Health Organization (WHO) declared COVID-19 as a pandemic. In order to contain SARS-CoV-2 diffusion and social transmission, many countries around the world, including Italy, implemented restriction measures, such as lockdown, which involved social distancing, the use of facemasks, abolished travels between countries and cities, and imposed smart working and distance learning on schools and universities (Cowling and Aiello 2020; Deserti et al. 2020).

Worldwide, the implemented restriction measures led to both positive and negative impacts on the environment. Some negative impacts were the impairment of waste recycling, the increase of medical waste production, and the overuse of disinfectants (Diffenbaugh et al. 2020; Facciolà et al. 2021). On the contrary, the situation caused also a positive effect reducing noise and air pollutant emissions (Kumar et al. 2020) which, in some world areas, resulted in significant changes in air quality in terms of pollutant concentrations (Addas and Maghrabi 2021; Agarwal et al. 2020; Shakoor et al. 2020; Sokhi et al. 2021).

In Europe, independently of the meteorological conditions, lockdown measures generally induced a decrease of NO_2_ which is generally released by vehicular traffic. Moreover, although to a lesser extent than NO_2_, these measures also reduced particulate matter (PM) concentrations in many countries (e.g., Spain, Turkey, and Croatia) (Briz-Redón et al. 2021; EEA 2020; Jakovljević et al. 2021; Menut et al. 2020; Orak and Ozdemir 2021; Tobías et al. 2020). However, in others, such as Italy, the PM concentrations remained unchanged or increased due to the lockdown in particular in urban sites located in the Padana Plain (Cameletti 2020; Gualtieri et al. 2020; Robotto et al. 2022; Volta et al. 2022). This discrepancy may be due to many factors that can affect the release, the dispersion, and the transformation of air pollutants such as emission sources, meteorological conditions, and geographic conformation. A great amount of urban PM_2.5_ is composed of secondary aerosol, which is generated in the air via oxidation, and a previous study highlighted that this secondary particle formation was often favored under lockdown (Robotto et al. 2022).

Although different studies have proven that the restriction measures (e.g., lockdown) are able to affect air quality changing PM_10_ and PM_2.5_ concentrations (EEA 2020) and chemical composition of PM (Altuwayjiri et al. 2021), to our knowledge, there are no studies on the impact of lockdown on biological effects induced by PM. PM is a complex mixture and it is constituted by different chemicals with a different toxicity. For examples, many different polycyclic aromatic hydrocarbons (PAHs) can be found in PM and some of them are classified as carcinogenic to humans (group 1, benzo(a)pyrene (BaP)) or probably/possibly carcinogenic to humans (group 2A/2B) by the International Agency for Research on Cancer (IARC 2010). Although PM toxicity may be evaluated measuring the concentrations of the toxic chemicals adsorbed on it, the overall biological effect of this complex mixture cannot be easily quantified using the concentrations of individual chemicals. Indeed, it is not feasible to measure the concentration of all the toxic chemicals considering also their metabolites and the toxicity of some chemicals may be still uncertain. Moreover, it is hard to define the biological effect of this complex mixture, since antagonistic and synergistic interactions can occur among the chemicals. Therefore, in addition to the quantification of toxic chemicals, the assessment of air quality in terms of toxicity can be performed using effect-based tools (Gea et al. 2021; Marangon et al. 2021). These tools are able to quantify the overall biological effect induced by chemical mixtures and are particularly suitable for complex mixtures with low concentrations of multiple chemicals (e.g., PM) (Marangon et al. 2021). Among the different effect-based tools that can be applied to assess the biological effects of air pollutants, the in vitro assays are widely applied also because they provide results in a short period. They are able to quantify different biological effects of PM such as cytotoxicity, genotoxicity, mutagenicity, and hormonal activity, giving insights into the potential health effects induced by different PM samples (Gea et al. 2021).

The aim of the study was to assess whether lockdown influenced biological effect of PM extracts in the Padana Plain (Piedmont region, North-West of Italy). The PM was daily collected during 2019 (pre-pandemic) and 2020 (containment measures and lockdowns) in four monitoring stations of the Regional Agency for Environmental Protection of Piedmont Region (ARPA Piemonte) characterized by different pollutant sources. The stations are part of a monitoring network which was designed by the Italian government in order to monitor the air quality as required by the European legislation (European Commission Directive 2008/50/EC, Italian Legislative Decree 155/2010). The filters were extracted with organic solvents and biological effects induced by PM extracts were assessed through in vitro assays. In particular, the cytotoxicity and the genotoxicity were assessed on BEAS-2B cells using the WST-1 assay and the Comet assay respectively, the mutagenicity was evaluated on TA98 and TA100 strains (± S9) with the Ames test, and the estrogenic activity was tested on MELN cells using a luciferase gene reporter assay. Furthermore, the concentrations of PM_10_, PM_2.5_, and four PAHs were analyzed.

The investigated area is characterized by peculiar meteorological/geographic conditions that are among the causes that induce high air pollutant concentrations (i.e., it is a “hot spot” for air quality in Europe) (Robotto et al. 2022). Here, many policy efforts were made in order to improve air quality–reducing air pollutant emissions but they were not always successful. Therefore, the peculiar situation of the lockdown, in which some air pollutant sources were blocked, was an interesting case study. Indeed, studying the potential change of air quality (in terms of PM concentrations, chemical composition, and biological effects) due to the lack of some pollutant sources could give also important suggestions for policy makers.

## Materials and methods

### PM sampling and extraction

PM sampling was performed in the Northwest of Italy in the four stations located (i) in an urban background site characterized by moderate traffic level (city of Torino in the Padana Plain—urban background site), (ii) in an urban traffic site characterized by high traffic level (city of Settimo T.se—urban traffic site), (iii) in a rural site characterized by low pollution levels (city of Dernice—rural site), and (iv) in a site located near an incinerator (city of Beinasco—incinerator site).

For each site, PM was daily collected on quartz-fibre filters (Ø = 47 mm) from 1 January 2019 to 31 December 2020 using low volume samplers (flow = 2.3 m^3^/h). In urban background, urban traffic, and rural sites, the PM_2.5_ was collected, while the PM_10_ was sampled in the incinerator site.

Daily filters of 2020 were weighted and pooled in six monthly pools for each site: January/February, March, April, May/June, July/August/September (summer), and October/November/December (autumn). These pools were selected considering the restrictive measures due to the SARS-CoV-2 pandemic implemented by the Italian government in 2020. In January and February, restrictive measures were not implemented. March and April were subjected to several strict containment measures like the closure of non-essential commercial activities and travel restrictions (first lockdown). Starting from May 2020, activities were gradually reopened and restrictions were almost absent during all summer. Finally, the restriction measures were re-introduced in autumn following a further increase of the number of infections and hospitalizations (second lockdown). Daily filters of 2019 (pre-pandemic period) were pooled as filters of 2020 for comparison.

Each pool was extracted through ultrasounds using acetone/cyclohexane (1:1) in order to collect organic-extractable compounds (Schilirò et al. 2016). Briefly, filters of each pool were cut in small pieces and washed three times with acetone/cyclohexane (1:1) using an ultrasonic water bath. Filters and solvent were then vortexed for 1 min and, in order to remove filter debris, they were centrifuged at 5000 r/min for 10 min. The supernatant was evaporated using a rotary evaporator, re-suspended in dimethyl sulfoxide (DMSO), and the extracts were stored at –20℃ until analysis (extract PM concentration = 20,000 m^3^/mL). A pool of blank filters was extracted and tested with all in vitro assays as experimental control. Even if the in vitro assays cannot exactly reproduce the real exposure, PM doses were selected in order to be similar to the real exposure (tested concentrations = 1 – 50 m^3^/mL for the human cell assays, 2.5 – 20 m^3^/plate for the Ames test; average breathed air by an adult in 1 day = 20 m^3^ (ECHA 2012)).

### Meteorological and air quality data

Meteorological data of the Piedmont Region were collected from the ARPA Piemonte annual reports (Regional Agency for Environmental Protection of Piedmont 2019, 2020), while pollution data of each sampling site were collected from the ARPA Piemonte website (Regional Agency for Environmental Protection of Piedmont 2022). The concentrations of PM_10_, PM_2.5_, and four PAHs (BaP, benzo(a)anthracene, benzo(b + j + k)fluoranthene and indeno(1,2,3-cd)pyrene) were analyzed. Data were processed according to the monthly pools applied for the PM extraction; therefore, for 2019 and 2020, the mean pollutant concentrations were calculated for January/February, March, April, May/June, July/August/September (summer), and October/November/December (autumn).

PAH concentrations were used to calculate the Toxic Equivalency Factor (TEF), which expresses the toxicity of PAH mixtures as BaP equivalents. Considering the carcinogenic potencies of PAHs in comparison to BaP (i.e., the reference PAH) (Nisbet and LaGoy 1992; Samburova et al. 2017), TEF was calculated as:

$$TEF=BAP\;concentration\;\times\;1+benzo\left(a\right)anthracene\;concentration\;\times\;0.1+benzo\left(b+j+k\right)fluoranthene\;concentration\;\times\;0.1+indeno\;\left(\mathrm{1,2},3-cd\right)\;pyrene\;concentration\;\times\;0.1$$

In order to calculate TEFs, the values below the limit of quantification (LOQ = 0.07 ng/m^3^) were considered equal to half the LOQ.

### Cytotoxicity

Cytotoxic effect induced by PM extracts was assessed on BEAS-2B cells using the WST-1 assay (Cell Proliferation Reagent WST-1, Roche). BEAS-2B, human bronchial epithelial cells, were obtained from the American Type Culture Collection (CRL-9609™). They were cultured in RPMI 1640 medium supplemented with phenol red, fetal bovine serum (FBS) (10% v/v), l-glutamine (4 mM), and penicillin–streptomycin (100 units/mL–100 µg/mL) (supplemented RPMI medium), at 37 °C and 5% CO_2_. The WST-1 assay was performed as previously described (Gea et al. 2021). Briefly, cells were seeded in 96-well plates in supplemented RPMI medium without phenol red (5000 cells/well, 100 µL/well) and exposed to PM organic extracts (equivalent to 10, 25, 50 m^3^/mL). After exposure (24 h or 72 h), WST-1 dye solution was added (10 µL/well) and the cells were incubated for 2/3 h. Finally, the absorbance of each well was measured at 440 nm (Infinite Reader M200 Pro, Tecan Trading AG, Switzerland). Cells exposed to DMSO were used as negative control. Data were expressed as percentages of cell viability with respect to negative control. All experiments were performed in quadruplicate (four wells for each experimental condition).

### Genotoxicity

Genotoxic effect induced by PM extracts was tested on BEAS-2B using the Comet assay (Bonetta et al. 2019; Collins et al. 2023; Tice et al. 2000). Cells were seeded in 6-well plates (300,000 cells/well, 1 mL/well) and exposed to PM organic extracts (range 10–50 m^3^/mL) in supplemented RPMI medium without FBS. The tested doses were selected considering the results of the cytotoxicity assay (WST-1 assay). In particular, extracts that induced a higher cytotoxic effect (cell viability % < 70%) were tested at lower doses than extracts that induced a lower cytotoxicity. In order to study a potential dose–response relationship, each sample was tested at the highest testable dose and at least at one intermediate dose. After exposure (24 h), cell viability was assessed using trypan blue dye and cells were embedded in low melting point agarose (0.7%) on slides. Slides were placed overnight in lysis solution (4 °C), immersed in an alkaline electrophoresis buffer (20 min), and subjected to electrophoresis (20 min, 1 V/cm, and 300 mA). Then, slides were neutralized, fixed, and dried. For the analysis of DNA damage, DNA was stained with ethidium bromide (20 µg/mL) and the percentage of tail intensity was quantified with Comet Assay IV analysis system (Perceptive Instruments, Instem, England) using a fluorescence microscope (Axioskop HBO 50, Zeiss, Italy). Cells exposed to DMSO were used as negative control, while 4-nitroquinoline N-oxide (1, 1.5, 2 mg/L) was used as positive control. All experiments were performed in duplicate (two gels for each experimental condition) and in each gel the % of tail intensity was quantified considering 50 cells. The fluorescence intensity obtained from the comet tail was used as an indicator of the amount of DNA damage. The results were reported according to the latest MIRCA guideline (Møller et al. 2020).

### Mutagenicity

The mutagenicity of the organic PM extracts was assessed through the Ames test (Maron and Ames 1983). Two *Salmonella typhimurium* strains (frameshift strain-TA98 and base-substitution strain-TA100) were exposed to PM organic extracts (equivalent to 2.5, 5, 10, 20 m^3^/plate) both with and without Aroclor-induced rat-liver homogenate activation (S9). After exposure (48 h), colonies were counted through an automatic colony counter (Protos, Synoptics, UK). The results were expressed as mutagenicity ratio per 20 m^3^ (MR) and total mutagenicity factor per 20 m^3^ (TMF). MR and TMF were calculated as:

$$MR=\frac{total\;revertants-spontaneous\;revertants}{spontaneous\;revertants}$$

$$TMF=MR\;TA98+MR\;TA98\;\left(+S9\right)+MR\;TA100+MR\;TA100\;(+S9)$$

Extracts were considered mutagen when they induced a MR ≥ 1. Strains exposed to DMSO were used as negative control, while 4-nitroquinoline N-oxide (0.5 µg/plate), methyl methane-sulfonate (0.25 µg/plate), and 2-aminoanthracene (2 µg/plate) were used as positive controls for TA98, TA100, and TA98 (+ S9)/TA100 (+ S9), respectively. All experiments were performed in duplicate.

### Estrogenic activity

The estrogenic activity of PM organic extracts was assessed on MELN cells using the luciferase gene reporter assay. MELN cells, human breast epithelial cells (MCF-7) transfected with the ERE-βGlob-Luc-SVNeo plasmid, were provided by Dr. P. Balaguer (INSERM, Montpellier, France). They were cultured in complete Dulbecco’s Modified Eagle’s Medium Nutrient Mixture F12-Ham supplemented with phenol red, FBS (5% v/v), l-glutamine (4 mM), and penicillin–streptomycin (100 units/mL–100 µg/mL), G418 (1 mg/mL) at 37 °C and 5% CO_2_. The luciferase gene reporter assay was performed as previously described using the One-Glo Luciferase Assay System (Promega) (Balaguer et al. 1999; Gea et al. 2021; Schilirò et al. 2012). Briefly, for 3 days, cells were adapted to the test medium (Dulbecco’s Modified Eagle’s Medium Nutrient Mixture F12-Ham without phenol red, supplemented with l-glutamine (4 mM), penicillin–streptomycin (100 units/mL–100 µg/mL), and 5% v/v of dextran-coated charcoal-treated FBS). Then, they were seeded in 96-well plates (40,000 cells/well, 100 µL/well) and exposed to PM organic extracts (range 1 – 50 m^3^/mL). After exposure (21 h), One-Glo Luciferase reagent was added (100 µL/well) and the luminescence was measured using a luminometer (Infinite Reader M200 Pro, Tecan, Switzerland). Cells exposed to DMSO were used as negative control, while cells treated with 17β-estradiol 10^–8^ M were used as positive control. Moreover, seven concentrations of 17β-estradiol from 10^–12^ to 10^–8^ M were used as a standard positive curve. The estrogenic activity was expressed as relative luciferase activity and it was calculated as percentage of activity induced by the treatment with respect to the activity of the positive control (relative luciferase activity of 17β-estradiol 10^–8^ M = 100%). The estrogenic activity of extracts was reported as 17β-estradiol equivalent concentration (EEQ) that was calculated using the concentrations of 17β-estradiol and extracts at which 50% of biological effect was achieved (EC50) through the formula:

$$EEQ=\frac{17\beta-estradiol\;EC50}{PM\;extract\;EC50}$$

The EC50 of 17β-estradiol and extracts was calculated by dose–response curves, which were estimated through a probit regression between the relative luciferase activity and Log transformed concentrations of 17β-estradiol or extracts.

All experiments were performed in quadruplicate (four wells for each experimental condition) and, in the experimental conditions, the detection limit was equal to 0.006 pg/m^3^.

### Statistical analysis

Statistical analysis was performed using IBM SPSS Statistics 27.0. The normal distribution of the data was assessed with the Shapiro–Wilk test.

Pollutant concentrations and mutagenicity and estrogenic activity results were analyzed using the Kruskal–Wallis test followed by pairwise comparisons with Bonferroni adjustment to compare the differences among the sites; the Mann–Whitney test was used to evaluate the differences between warm and cold months while the Wilcoxon test was used to compare 2019 and 2020 data.

Cytotoxicity results were analyzed using the Kruskal–Wallis test followed by Dunnett’s post hoc test in order to assess a significant cytotoxic effect induced by PM extracts with respect to the negative control. Moreover, the Wilcoxon test was applied in order to compare 2019 and 2020 cytotoxicity data.

Genotoxicity results were analyzed using the one-way ANOVA test followed by Dunnett’s post hoc test in order to assess a significant genotoxic effect induced by PM extracts with respect to the negative control. Moreover, the *T*-test for paired samples was applied in order to compare 2019 and 2020 genotoxicity data.

Results were considered statistically significant with *p*-value ≤ 0.05.

## Results

### Meteorological and pollutant data

The meteorological conditions measured during 2019 and 2020 in the Piedmont region are reported in Table 1. The year 2019 was characterized by higher numbers of windy days and rainy days than 2020. Moreover, also the mean annual temperature (℃) and precipitation (mm) were higher in 2019 than in 2020.

**Table 1:** Meteorological conditions during 2019 and 2020 in the Piedmont Region

Meteorological conditions	2019	2020
No. of days characterized by Foehn wind	86	62
No. of days with rainfall (at least 1-mm precipitation)	100	69
Mean annual atmospheric precipitation (mm)	1227	838
Mean annual temperature in Torino (°C)	14.3	14.0

The concentrations of air pollutants (PM_10_, PM_2.5_, BaP, and TEF) in the four selected sites are reported in Table 2 divided by monthly pool and year. Concentrations of PM_10_ and PM_2.5_ were statistically different among sites; indeed, rural site showed significantly lower PM_10_ and PM_2.5_ concentrations than the concentrations measured in the other three sites (PM_10_: rural site vs*.* urban background site *p* ≤ 0.05 rural site vs*.* urban traffic/ incinerator sites* p* ≤ 0.001; PM_2.5_: rural site vs. urban background/ urban traffic/incinerator sites *p* ≤ 0.05). On the contrary, no significant difference was found regarding BaP and TEF concentrations among sites.

**Table 2:** Air pollutant concentrations during 2019 and 2020 in the four sites divided by monthly pools

Air pollutant concentrations	Period	Urban background site		Urban traffic site		Rural site		Incinerator site	
		2019	2020	2019	2020	2019	2020	2019	2020
PM_10_ (µg/m^3^)	January/ February	53.3	58.1	72.8	66.5	13.6	14.5	52.9	53.5
	March	23.6	24.6	32.7	32.3	9.3	13.1	26.4	24.9
	April	15.2	14.8	19.0	21.3	9.6	12.5	20.2	19.1
	May/June	14.1	11.4	17.4	15.0	11.7	9.7	16.3	12.8
	Summer (Jul, Aug, Sept)	19.3	16.0	25.5	19.7	14.6	13.3	18.4	16.1
	Autumn (Oct, Nov, Dec)	30.0	40.8	34.4	44.0	10.7	11.0	28.1	38.2
PM_2.5_ (µg/m^3^)	January/ February	38.1	43.5	49.4	48.8	10.9	12.5	42.4	42.6
	March	14.0	18.3	18.5	21.7	6.9	8.8	16.8	18.5
	April	10.9	9.6	11.1	13.0	7.0	8.6	14.0	13.4
	May/June	8.9	7.7	9.1	8.2	8.4	6.6	10.2	8.4
	Summer (Jul, Aug, Sept)	13.8	11.0	13.4	9.6	11.6	9.2	11.8	10.4
	Autumn (Oct, Nov, Dec)	23.4	31.2	25.5	34.6	8.1	7.9	21.1	29.3
BaP (ng/m^3^)	January/ February	1.90	2.00	3.05	2.90	0.10	0.07	2.05	1.80
	March	0.20	0.40	0.40	0.70	< LOQ	0.10	0.30	0.40
	April	< LOQ	0.10	0.10	0.20	< LOQ	< LOQ	< LOQ	0.10
	May/June	< LOQ	< LOQ	< LOQ	< LOQ	< LOQ	< LOQ	< LOQ	< LOQ
	Summer (Jul, Aug, Sept)	0.06	< LOQ	< LOQ	< LOQ	< LOQ	< LOQ	< LOQ	< LOQ
	Autumn (Oct, Nov, Dec)	0.77	1.07	1.13	1.60	0.28	0.06	0.70	1.03
TEF (ng/m^3^)	January/ February	2.70	2.82	4.26	3.99	0.17	0.14	2.89	2.55
	March	0.31	0.58	0.66	1.01	0.06	0.14	0.47	0.61
	April	0.09	0.14	0.16	0.29	0.05	0.06	0.09	0.16
	May/June	0.05	0.06	0.06	0.05	0.05	0.05	0.06	0.05
	Summer (Jul, Aug, Sept)	0.08	0.05	0.05	0.06	0.05	0.05	0.05	0.05
	Autumn (Oct, Nov, Dec)	1.08	1.51	1.57	2.21	0.39	0.12	1.00	1.45

< *LOQ*, below the quantification limit (LOQ = 0.07 ng/m^3^)

In order to calculate TEF, values below the LOQ were considered equal to half the LOQ

The concentrations of all the air pollutants were higher in cold months (January/February, March, and autumn) with respect to warm months (April, May/June, summer) (*p* ≤ 0.05) in each site, with the exception of rural site that showed low concentrations of PM_10_ and PM_2.5_ in both warm and cold months.

Finally, comparing the concentrations of 2019 vs. concentrations of 2020 divided by site, no statistically significant difference was found between 2019 and 2020 air pollutants in each site. Moreover, the same result was obtained comparing concentrations of the four sites of 2019 with the concentrations of the four sites of 2020 divided by monthly pool.

### Cytotoxicity

The results of the WST-1 assay showed that the organic extracts of PM collected in rural site did not induce any cytotoxic effect on BEAS-2B cells with respect to negative control both in 2019 and 2020 (data reported in Figs. S1 and S2, Appendix A). Moreover, also the April, May/June, and summer extracts of the other three sites did not induce any cytotoxicity (data reported in Fig. S3, Appendix A). On the contrary, a significant effect was induced by some extracts of the urban background, urban traffic, and incinerator sites sampled in January/February, March, and autumn. These results highlighted a seasonal difference in the cytotoxic effect (i.e., higher cytotoxicity in cold months with respect to warm months).

**Fig. 1:**
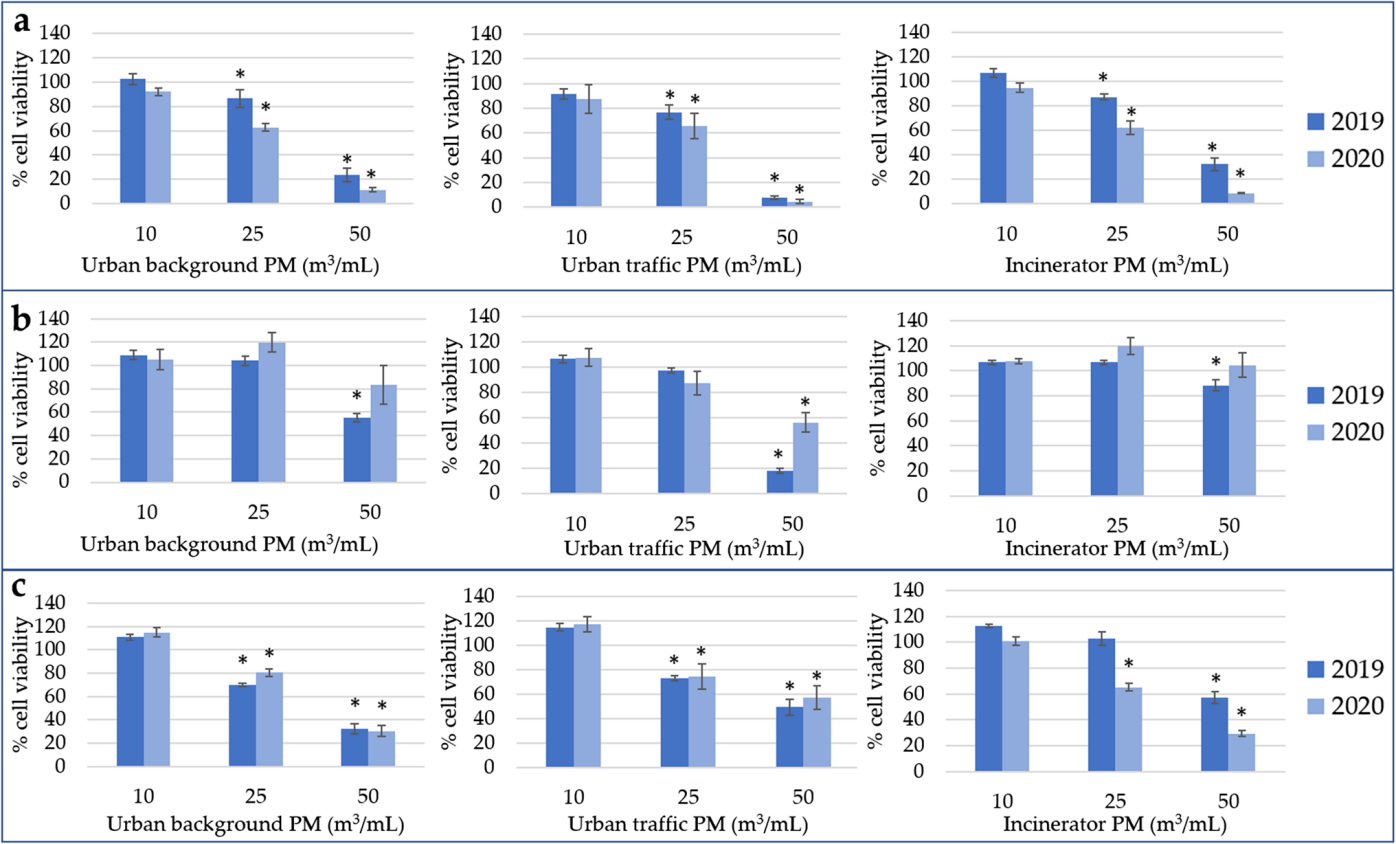
Cytotoxicity of particulate matter (PM) extracts at 24 h (data at 72 h are shown in Fig. S4 in Appendix A): **a** January/February PM extracts (no restrictions), **b** March extracts (first lockdown, hard restrictions); **c** autumn PM extracts (second lockdown, intermediate restrictions). April, May/June, and summer PM extracts and all the rural PM extracts (2019 and 2020, 24 h and 72 h) did not induce any cytotoxic effect (data shown in Figs. S1, S2, S3 in Appendix A). Data are expressed as means ± standard deviations. *p ≤ 0.05 Kruskal–Wallis test followed by Dunnett’s post hoc test vs. negative control (% cell viability of negative control = 100%)

Figure 1a and Fig. S4a (Appendix A) show the effect induced after 24-h and 72-h exposures, respectively, by January/February extracts (no restrictions). The extracts of urban background, urban traffic, and incinerator sites induced a significant cytotoxic effect at 25 and 50 m^3^/mL for both the exposure times (24 h and 72 h) and in both years (2019 and 2020). Moreover, the comparison between 2019 and 2020 data showed that in each site (urban background, urban traffic, and incinerator sites) for each exposure time, the 2020 extracts induced a significantly higher cytotoxic effect than the 2019 extracts.

Figure 1b and Fig. S4b (Appendix A) show the effect induced after 24-h and 72-h exposures, respectively, by March extracts (first lockdown: several strict containment measures). The 2019 extracts of urban background, urban traffic, and incinerator sites induced a significant cytotoxic effect at 50 m^3^/mL after both the exposure times (24 h and 72 h). On the contrary, no effect was induced at all the tested doses by the 2020 extracts of urban background and incinerator sites, showing that the cytotoxicity was lower in 2020 than in 2019. The 2020 extract of the urban traffic site (similarly to the 2019 extract) induced a significant cytotoxic effect at 50 m^3^/mL after both the exposure times; however, the mean percentage of cell viability of the 2020 extract was higher than the mean percentage of the 2019 extract. Although the difference was not significant, the result suggests a higher cytotoxic effect in 2019 than in 2020, partially confirming the results obtained testing the urban background and the incinerator extracts.

Figure 1c and Fig. S4c (Appendix A) show the effect induced after 24-h and 72-h exposures, respectively, by autumn extracts (second lockdown: intermediate restrictions). The extracts of urban background, urban traffic, and incinerator sites induced a significant cytotoxic effect at 25 and 50 m^3^/mL for both the exposure times (24 h and 72 h) and in both years (2019 and 2020), with the exception of 2019 incinerator autumn which induced a significant effect only at the highest tested dose. The comparison between 2019 and 2020 data showed that the cytotoxicity induced by urban background and urban traffic sites after the 72-h exposure was significantly higher in 2019 than in 2020, while on the contrary, the cytotoxicity induced by incinerator samples was significantly lower in 2019 than in 2020 at both the exposure times (24 h and 72 h).

### Genotoxicity

The results of the Comet assay showed that the May/June and summer extracts of all sites did not induce any genotoxic effect on BEAS-2B cells with respect to negative control both in 2019 and 2020 (data reported in Figs. S5 and S6, Appendix A). On the contrary, a significant effect was induced by some extracts collected in January/February, March, April, and autumn. Similar to the cytotoxicity results, these results highlighted a seasonal difference in the genotoxic effect (i.e., higher genotoxicity in cold months with respect to warm months).

**Fig. 2:**
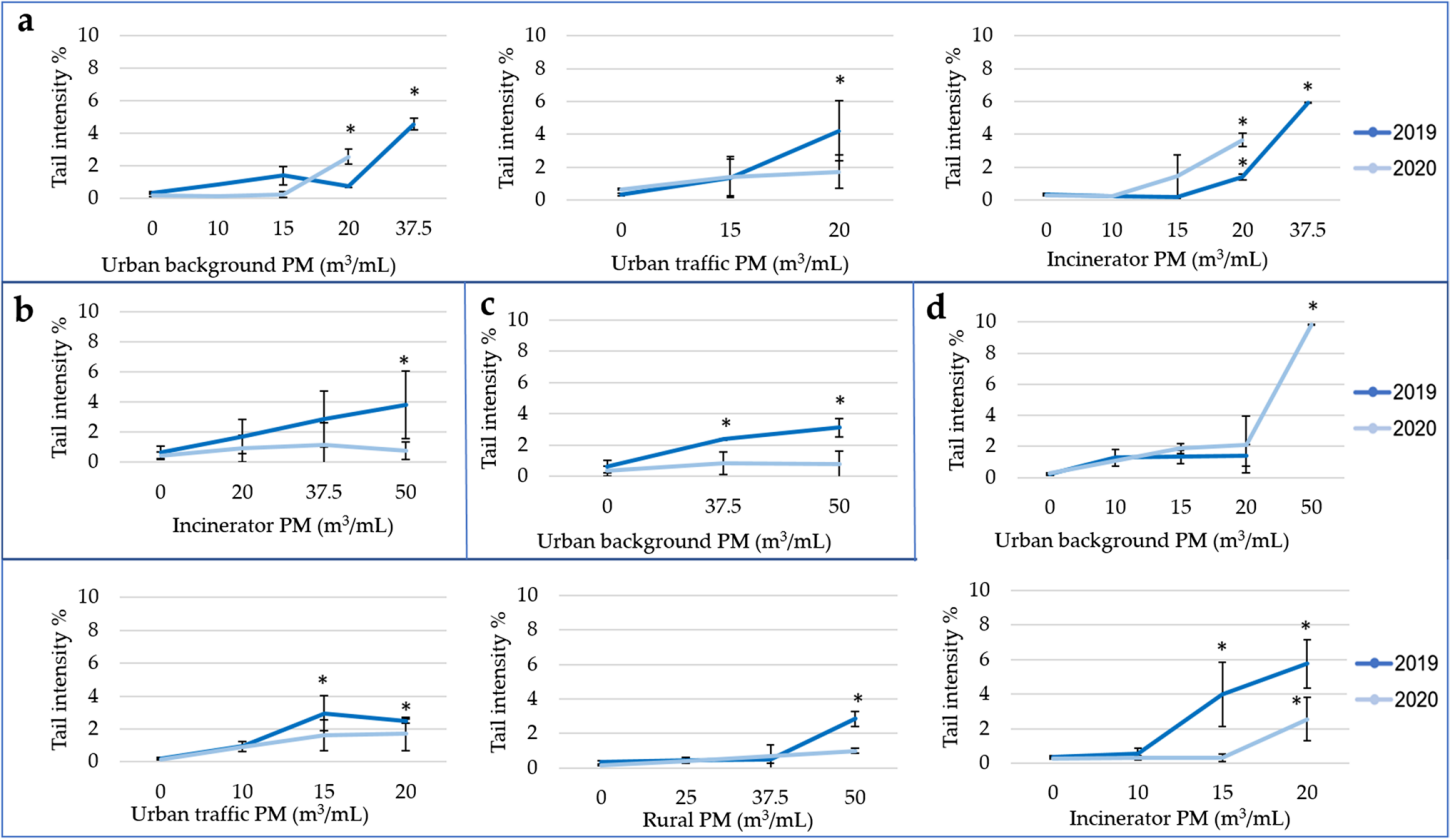
Genotoxicity of particular matter (PM) extracts at 24-h exposure: **a** January/February PM extracts (no restrictions), **b** March PM extracts (first lockdown, hard restrictions); **c** April PM extracts (first lockdown, hard restrictions); **d** autumn PM extracts (second lockdown, intermediate restrictions). The results of the PM extracts that did not induce any genotoxic effect are reported in Figs. S5 – S9 in Appendix A. Data are expressed as means ± standard deviations. *p ≤ 0.05 one-way ANOVA test followed by Dunnett’s post hoc test vs. negative control

Figure 2a shows the effect induced by January/February extracts (no restrictions). In 2019 and 2020, the urban background extract induced a significant genotoxic effect with respect to negative control at the highest tested doses (37.5 m^3^/mL and 20 m^3^/mL, respectively) (*p* ≤ 0.001). Considering the urban traffic extracts, only the 2019 extract induced a significant effect at the highest tested dose (20 m^3^/mL) (*p* ≤ 0.05). Regarding the genotoxic effect of incinerator extracts, a significant increase of DNA damage with the increase of the dose was observed for the 2019 extract starting from 20 m^3^/mL dose (*p* ≤ 0.001), while the 2020 extract induced a genotoxic damage only at the highest tested dose (20 m^3^/mL) (*p* ≤ 0.05). Finally, no genotoxic effect was shown exposing cells to rural extracts (data reported in Fig. S7, Appendix A).

The comparison between 2019 and 2020 data showed that the urban traffic extract induced a significantly higher effect in 2019 than in 2020, while the urban background and incinerator extracts showed an opposite trend with a higher effect in 2020 (*p* ≤ 0.05).

Figure 2b shows the genotoxic effect induced by March extracts (first lockdown: several strict containment measures). All the extracts did not induce any statistically significant effect with respect to negative control in both years (data reported in Fig. S8, Appendix A), with the exception of the 2019 incinerator extract, which induced a significant genotoxic effect at the highest tested dose (50 m^3^/mL) (*p* ≤ 0.05).

The comparison between 2019 and 2020 data showed that, in the month of March, a significant difference was detected between 2019 and 2020 extracts only for the incinerator site (*p* ≤ 0.05). In particular, a higher DNA damage was induced by 2019 extract.

Figure 2c shows the genotoxic effect induced by April extracts (first lockdown: several strict containment measures). Only the 2019 extract of the urban background site showed a significant increase of DNA damage with the increase of the dose (*p* ≤ 0.05) starting from the lowest tested dose (37.5 m^3^/mL), while the other extracts did not cause any genotoxic effect (data reported in Fig. S9, Appendix A).

The comparison between 2019 and 2020 data showed that, in the month of April, a significant difference was detected between 2019 and 2020 extracts only for the urban background site (*p* ≤ 0.05). In particular, a higher DNA damage was induced by 2019 extract.

Figure 2d shows the effect induced by autumn extracts (second lockdown: intermediate restrictions). Considering the urban background extracts, no genotoxic effect was induced by 2019 extract while the 2020 extract induced a significant genotoxic effect at the highest tested dose (50 m^3^/mL) (*p* ≤ 0.001). Moreover, regarding urban traffic extracts, an increase of DNA damage was observed for the 2019 extract with respect to negative control starting from 15 m^3^/mL (*p* ≤ 0.05), while no effect was found for the 2020 extract. The 2019 rural extract induced significant genotoxic effect at the highest tested dose (50 m^3^/mL) (*p* ≤ 0.001), while no genotoxic effect was observed for the 2020 rural extract. Finally, in the incinerator site, a significant increase of DNA damage with the increase of the dose was observed testing the 2019 extract (*p* ≤ 0.05), while the 2020 extract induced a genotoxic damage only at the highest tested dose (20 m^3^/mL) (*p* ≤ 0.05).

Comparing the genotoxic effect between 2019 and 2020, in 3 out of 4 sites (urban traffic, rural, and incinerator sites), a higher effect was induced by 2019 extracts with respect to 2020 ones.

### Mutagenicity

**Fig. 3:**
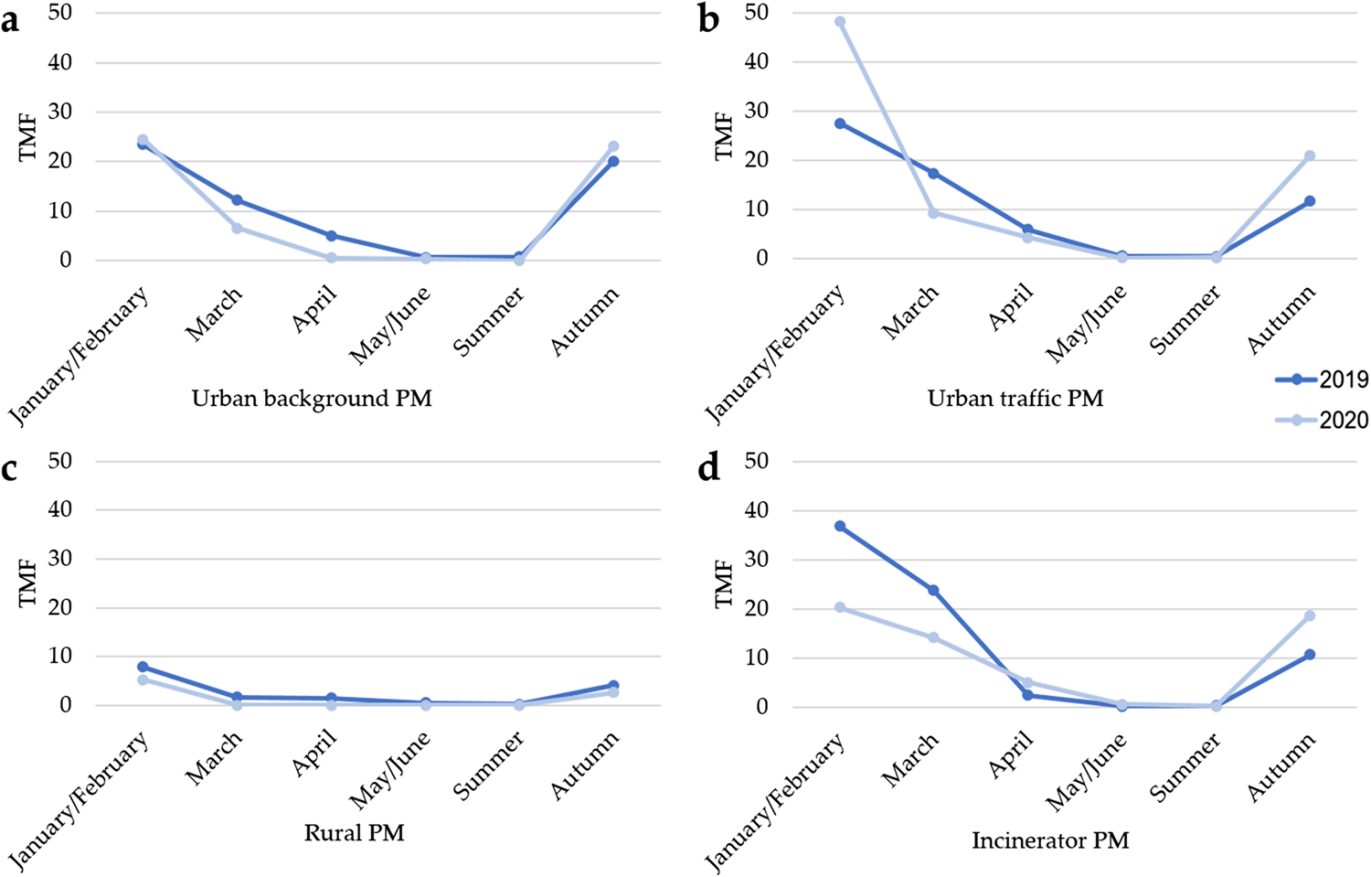
Total mutagenicity factors (TMFs) of 2019 and 2020 particular matter (PM) organic extracts: **a** urban background PM extracts, **b** urban traffic PM extracts, **c** rural PM extracts, **d** incinerator PM extracts

In Fig. 3, the mutagenicity results are reported, while in Table S1 (Appendix A) the number of strains on which the extracts induced a significant mutagenic effect (MR ≥ 1) are reported divided by year and site.

No differences were found in TMF values among sites, although the lowest TMF values were induced by the extracts collected in the rural site. Moreover, the majority of the extracts collected in this site did not induce any significant mutagenic effect on any of the tested strains (TA 98, TA98 + S9, TA100, TA100 + S9), suggesting a lower mutagenicity of this rural site with respect to the mutagenicity of the other sites.

The TMF values of each site were significantly higher in cold months (January/February, March, and autumn) with respect to warm months (April, May/June, summer) (*p* ≤ 0.05) that generally did not induce any significant mutagenic effect on all the tested strains.

Finally, comparing the PM mutagenicity between 2019 and 2020, in each site no statistically significant difference was found among the MRs of TA98, TA98 + S9, TA100, and TA100 + S9 divided by monthly pools. Although no statistically significant difference was found considering the MRs, in March (first lockdown), the TMFs of the urban background, urban traffic, and incinerator sites were lower in 2020 than in 2019. The same trend was shown also testing the urban background extracts collected in April (first lockdown) and the incinerator extracts collected in January/February (no restrictions). An opposite trend (i.e., higher mutagenicity in 2020 than in 2019) was found for the urban traffic extracts collected in January/February (no restrictions) and the urban traffic and the incinerator extracts collected in autumn (second lockdown).

### Estrogenic activity

**Fig. 4:**
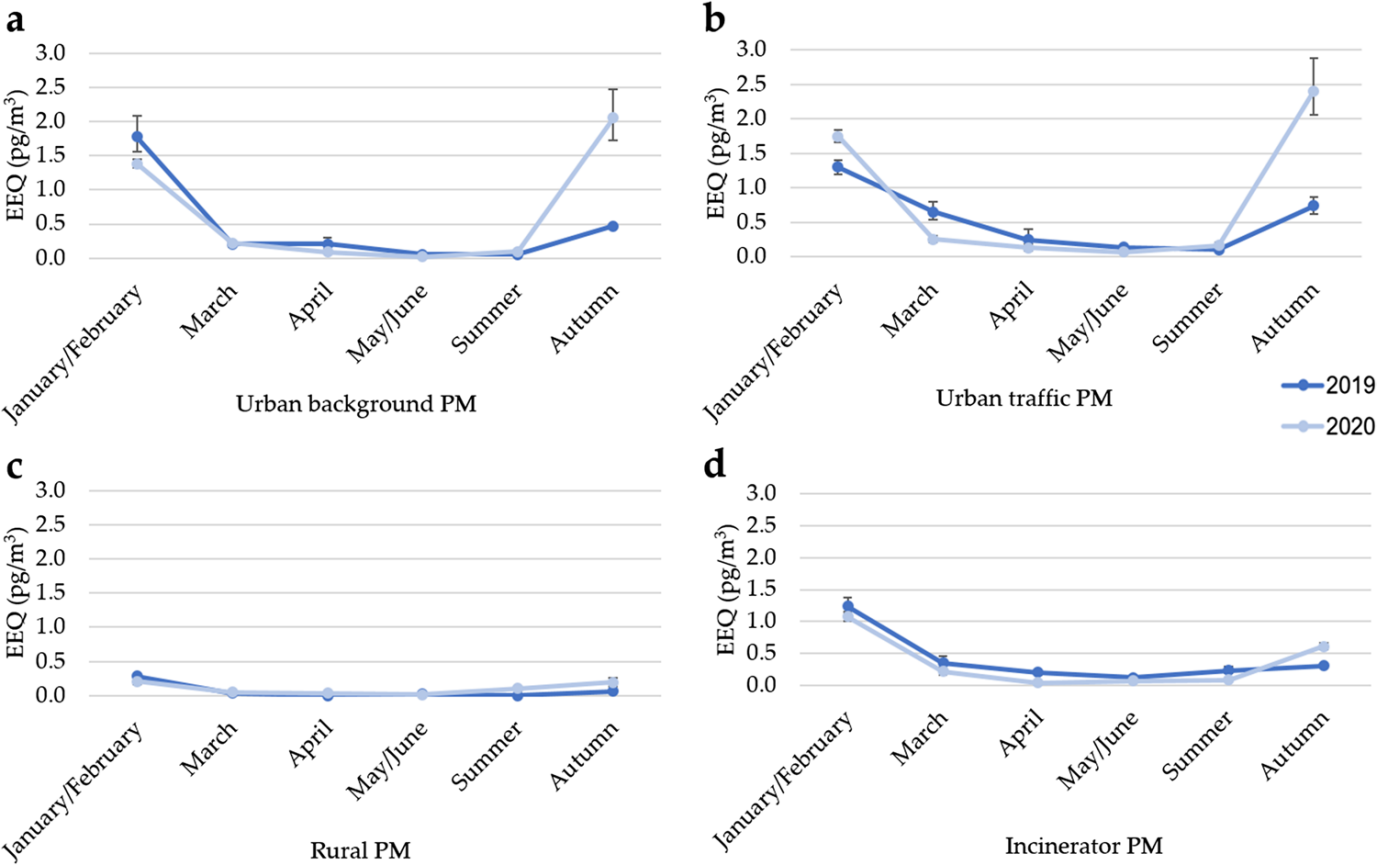
Estrogenic activity of 2019 and 2020 particulate matter (PM) organic extracts: **a** urban background PM extracts, **b** urban traffic PM extracts, **c** rural PM extracts, **d** incinerator PM extracts. Data are expressed as 17β-estradiol equivalent concentrations (EEQs) ± 95% confidence intervals

Figure 4 shows the results of the gene reporter luciferase assay. All samples induced a significant estrogenic activity with the exception of the rural extract of April 2019 and the rural extract of summer 2019 (reported as a half of detection limit). Overall, the EEQs ranged from 0.02 to 2.41 pg/m^3^. EEQs were statistically different among sites; indeed, the rural site showed significantly lower EEQs than the EEQs of the urban traffic and incinerator sites (rural site vs*.* urban traffic site *p* ≤ 0.05, rural site vs. incinerator site *p* ≤ 0.05). In all sites, the EEQs were higher in cold months (January/February, March, and autumn) with respect to warm months (April, May/June, summer) (*p* ≤ 0.05).

Finally, comparing the EEQs of 2019 vs. EEQs of 2020 divided by site, no statistically significant difference was found between 2019 and 2020 estrogenic activity in each site. Moreover, the same result was obtained comparing the EEQs of the four sites of 2019 with the EEQs of the four sites of 2020 divided by monthly pool. Although no statistically significant difference was found comparing 2019 vs*.* 2020 EEQs, a higher EEQ was found in March 2019 than in March 2020 (first lockdown) in the urban traffic site, while in the same site higher EEQs were detected in January/February 2020 (no restriction) than in 2019. Finally, extracts of Autumn 2020 (second lockdown) showed a higher estrogenic activity than extracts of Autumn 2019 especially in the urban background and the urban traffic sites.

## Discussion

In 2020, the SARS-CoV-2 virus and COVID-19 have spread at a global level. As a result of the increase in severe cases of COVID-19 and the consequent global health crisis, many countries implemented containment measures including lockdown periods to decrease social transmission of virus. These containment measures brought to many changes in movements, commercial activities, and industrial processes which potentially may have resulted in some effects on air pollutant concentrations and on PM biological effects.

Regarding pollutant concentrations, the results of the present study showed that overall PM_10_ and PM_2.5_ concentrations were significantly lower in the rural site than in all the other ones (urban background, urban traffic, and incinerator sites) demonstrating that PM concentrations are much higher in areas characterized by higher population density, vehicular traffic, and incinerator emissions than in rural areas. This result is not surprising; indeed, vehicular traffic, domestic heating, and industries can be considered among the main sources of PM and all of them, especially vehicular traffic, are lower in rural areas, characterized by a lower population density and less truck traffic, with respect to urban background, urban traffic, and industrial ones (Du et al. 2021; Juda-Rezler et al. 2020). At the European level, the EEA (2020) showed similar results with regard to PM concentrations levels in rural areas.

Pollutant results showed a seasonal trend of PM concentrations in almost all the sites in both 2019 and 2020, with the exception of the rural site which was characterized by low PM concentrations for all months of both 2019 and 2020. PM concentrations of urban background, urban traffic, and incinerator sites were higher in cold months (autumn and winter seasons) and lower in warm months (spring and summer seasons). In accordance with the present study, a seasonal trend of PM concentrations was reported by the study of Juda-Rezler et al. (2020) in an urban site located in Warsaw during 2016. Moreover, also other studies performed in the same area of the present one showed a comparable trend (Alessandria et al. 2014; Bonetta et al. 2019; Schilirò et al. 2015). This trend can be explained considering that Northwest Italy (Padana Plain) is an area with diffused air pollution, where during the cold season the geographical conformation and climatic conditions, as thermal inversion, favor pollutant accumulation rather than dispersion (Perrino et al. 2014; Schilirò et al. 2015). On the contrary, a sharp decrease in PM concentrations is generally reported during the warm season, when some emissions sources (such as domestic heating) are lacking (Schilirò et al. 2015) and meteorological conditions (such as wind speed, planetary boundary layer height, and atmospheric pressure) favor a higher pollutant dispersion (Robotto et al. 2022).

The results on TEF values showed that, despite a statistically significant difference among sites was not observed, the rural site was characterized by the lowest TEF values, while the urban traffic site showed the highest ones. This evidence confirmed that high traffic urban areas with higher population density are more polluted than rural ones. Similar to PM concentrations, TEF values were lower in warm months than in the cold ones. The decrease of TEFs in warm months can be attributed not only to the lack of some PAH sources, such as domestic heating, but also to a greater effect of pollutant photolysis (Schilirò et al. 2015). Indeed, during summer, high temperature and sunlight are favorable to PAH photochemical reactivity, reducing PAH concentrations and forming other secondary pollutants (Perrone et al. 2010).

In the present study, pollutant concentrations (PM_10_, PM_2.5_, BaP, and TEF) were comparable between 2019 (pre-pandemic) and 2020 (containment measures and lockdowns). This result suggested that, despite the containment measures and lockdowns causing a decrease in pollution emission by many pollutant sources (such as traffic and industrial activities), these measures did not strongly influence pollutant concentrations in this area. This result is in contrast with the data reported in Europe where COVID-19 restrictions led to a general reduction of air pollutant emissions and caused an air quality improvement in 2020 (Briz-Redón et al. 2021; Collivignarelli et al. 2020; Orak and Ozdemir 2021; Tobías et al. 2020) as reported also out of Europe, in particular, in the USA (Berman and Ebisu 2020; Liu et al. 2021), India (Gouda et al. 2021; Sharma et al. 2020; Shehzad et al. 2021), Korea (Ju et al. 2021), Malaysia (Abdullah et al. 2020), Brazil (Dantas et al. 2020), Thailand (Wetchayont 2021), and all over the world (He et al. 2021).

This unexpected result could be due to different factors. Indeed, even if the Italian containment measures reduced the vehicular traffic and industrial emissions (unnecessary travels were not allowed and non-essential industrial and commercial activities were closed), other emission sources such as agricultural and natural ones were probably not affected by the lockdown and, in addition, others could have been even increased by it. Indeed, people were forced to stay home, so there might have been an increase in PM emissions due to domestic heating (e.g., increase in biomass combustion) (EEA 2020). This explanation is supported by the study of Gualtieri et al. (2020), which assessed the change in air pollutant concentrations during the first Italian lockdown in six Italian cities and found that PM levels decreased to a lesser extent than expected and, in some cities, PM levels even increased during lockdown. Gualtieri et al. (2020) stated that the lockdown reduced the road traffic emissions as well as emissions of secondary aerosol precursors such as NOx and SO_2_; however, these reductions may have been counterbalanced by an increase of residential emissions (e.g., home heating, biomass combustion) and by an increase of agricultural ammonia emissions (ammonia is a precursor of secondary pollutants such as PM). Moreover, this explanation is also supported by the analysis of pollutant emission reported by ARPA (Regional Agency for Environmental Protection of Piedmont 2020) and Robotto et al. (2022). Indeed, they estimated that during the first lockdown (March and April 2020) primary PM_10_ emissions were comparable with the mean primary PM_10_ emissions generally released in this period because the reduction in the contribution from industry and traffic was counterbalanced by an overall emission increase from domestic heating. Finally, another explanation for the lack of PM reduction during lockdown may be that, in that period, waste recycling programs were suspended and disposable objects were more used (e.g., masks, gloves, take-away food packages, shopping boxes for home delivery) resulting in the increase of domestic and medical waste (Zambrano-Monserrate et al. 2020) that could have increased pollutant emissions from waste disposal plants.

In addition to PM sources, also meteorological conditions could have affected PM concentrations causing a lack of reduction. Indeed, considering interactions between meteorology, transport, chemical transformation, and dispersion of pollutants, ARPA estimated that the number of days favorable to PM_10_ formation was higher in 2020 than in 2019 (123 days in 2020, 96 days in 2019) and it reported that, due to meteorological conditions, the secondary PM was even increased during 2020 with respect to 2019 (Regional Agency for Environmental Protection of Piedmont 2020).

Considering the results of all the biological assays (cytotoxicity, genotoxicity, mutagenicity, estrogenic activity), a different biological effect was induced by the four sites. In particular, the lowest effect was induced by the rural site which is characterized by low traffic and low industrial/commercial activities. This result confirmed that the air quality of this site (in terms of PM_10_, PM_2.5_, and TEF values) is better than that of the other sites.

Moreover, the results of all the biological assays generally showed a seasonal trend comparable to the trend found for air pollutants (PM_10_, PM_2.5_, and TEFs). Indeed, high effects were induced by the extracts of cold monthly pools (January/February, March, autumn) while slight or no effects were induced by the extracts of warm monthly pools (April, May/June, summer). Previous studies showed that PM organic extracts collected in the Piedmont Region (i.e., the area of the present study) are able to induce biological effects with a seasonal trend. Indeed, Alessandria et al. (2014) using the LDH assay found that organic extracts of PM_10_ collected in Torino showed a cytotoxic effect with a seasonal trend. Moreover, a seasonal trend was found by Schilirò et al. (2015) and Bonetta et al. (2019) testing the genotoxicity of PM_10_ and PM_0.5_ organic extracts collected in Torino. Finally, in the study of Marangon et al. (2021), the organic extracts of PM_2.5_ collected in nine sites of the Piedmont Region showed a mutagenic effect with a seasonal trend. These trends are probably related to the higher level of PM and chemical pollutants in winter/autumn with respect to spring/summer confirming that, in the investigated area (Padana Plain), PM concentration, and its composition and its biological effects are strongly influenced by seasonal meteorological conditions.

Regarding the comparison between the biological effects induced by 2019 extracts and the effects induced by 2020 extracts, different results were found analyzing the different biological endpoints (Table 3). When the restrictive measures were not implemented yet (in January/February), the urban background, urban traffic, and incinerator extracts were more cytotoxic in 2020 than in 2019, while during March and autumn almost all the extracts collected in these sites induced a lower effect in 2020 (first and the second lockdowns) than in 2019 (pre-pandemic periods). This result suggested that, despite air pollutant concentrations not influenced by COVID-19 restrictions, these measures were able to decrease the cytotoxic effect of PM organic extracts in the urban background, urban traffic, and incinerator sites. Among March and autumn extracts of these sites, an opposite result was found only for the autumn extracts of the incinerator site. Indeed, in this site, the cytotoxic effect of PM collected during autumn was higher testing the 2020 extract than the 2019 one. Considering that the site is located near an incinerator, this unexpected result could be related to the fact that the reduction of the PM cytotoxic effect due to the decrease of traffic and industrial activities may have been counterbalanced by a higher combustion rate of waste during the pandemic which could have increased the cytotoxic effect of PM in this site.

**Table 3:** Comparison between the biological effects induced by 2019 extracts with respect to the effects induced by 2020 extracts

		Biological effects			
Period	PM extract	Cytotoxicity	Genotoxicity	Mutagenicity	Estrogenic activity
January/February (no restriction)	Urban background	2020 > 2019*	2020 > 2019*	2019 = 2020	2019 = 2020
	Urban traffic	2020 > 2019*	*2019* > 2020*	**2020 > 2019**	**2020 > 2019**
	Rural	2019 = 2020	2019 = 2020	2019 = 2020	2019 = 2020
	Incinerator	2020 > 2019*	2020 > 2019*	*2019* > *2020*	2019 = 2020
March (first lockdown, high restrictions)	Urban background	*2019* > 2020*	2019 = 2020	*2019* > *2020*	2019 = 2020
	Urban traffic	*2019* > *2020*	2019 = 2020	*2019* > *2020*	*2019* > *2020*
	Rural	2019 = 2020	2019 = 2020	2019 = 2020	2019 = 2020
	Incinerator	*2019* > 2020*	*2019* > 2020*	*2019* > *2020*	2019 = 2020
April (first lockdown, high restrictions)	Urban background	2019 = 2020	*2019* > 2020*	*2019* > *2020*	2019 = 2020
	Urban traffic	2019 = 2020	2019 = 2020	2019 = 2020	2019 = 2020
	Rural	2019 = 2020	2019 = 2020	2019 = 2020	2019 = 2020
	Incinerator	2019 = 2020	2019 = 2020	2019 = 2020	2019 = 2020
Autumn (second lockdown, intermediate restrictions)	Urban background	*2019* > 2020*	2020 = 2019	2019 = 2020	**2020 > 2019**
	Urban traffic	*2019* > 2020*	*2019* > 2020*	**2020 > 2019**	**2020 > 2019**
	Rural	2019 = 2020	*2019* > 2020*	2019 = 2020	2019 = 2020
	Incinerator	2020 > 2019*	*2019* > 2020*	**2020 > 2019**	2019 = 2020

Italic values: higher biological effects in 2019 than in 2020

Bold values: higher biological effects in 2020 than in 2019

^*^The difference was statistically significant

Similar to the cytotoxicity results, in January/February, the urban traffic extract showed a higher genotoxic effect in 2019 than in 2020 while, on the contrary, urban background and incinerator extracts showed an opposite trend with a higher effect in 2020. Since there were no restrictions during this period, the differences between 2019 and 2020 could be associated to differences in PM chemical composition which can be due to local variations of pollution sources between the two years.

Comparing the genotoxic effect of the first lockdown (March and April) between 2019 and 2020 extracts, a lower genotoxicity was highlighted in 2020 than 2019 in 2 samples (March from the incinerator site and April from the urban background site), confirming that the implementation of the pandemic restrictions decreased the PM biological effect in some sites. Moreover, in autumn (second lockdown), three sites (urban traffic, incinerator, and rural sites) highlighted a lower genotoxic effect in 2020 respect to 2019. Considering that in this period intermediate levels of restrictions were present, the lower genotoxic damage reported for these sites was probably related not only to the modified pollutant emissions related to lockdown but also to other factors (e.g., differences in meteorological condition or in PM chemical composition related to local variation of pollution sources between the two years).

In contrast with the cytotoxicity and the genotoxicity results, the results of the Ames test and the gene reporter assay did not show any statistically significant difference between 2019 and 2020. Although these results were not statistically significant, their trend was sometimes in accordance with the results of the other in vitro assays. Indeed, similar to the cytotoxicity results, the mutagenicity of the urban background, urban traffic, and incinerator sites in March was lower in 2020 (first lockdown) than in 2019. Moreover, similarly to the genotoxicity results, the urban background extract collected in April 2020 (first lockdown) was less mutagenic than the extract collected in April 2019. Finally, in accordance with the cytotoxicity results, a higher mutagenicity in 2020 than in 2019 was found for the urban traffic extracts collected in January/February (no restrictions) and the incinerator extracts collected in autumn (second lockdown). Regarding the estrogenic activity, the comparison between the effect induced by 2019 and 2020 extracts confirmed that the biological effect was lower in the urban traffic site during March 2020 (first lockdown) with respect to March 2019. Moreover, similar to the results of the other assays, the gene reporter assay showed that in January/February and autumn the biological effects increased in 2020 with respect to 2019 in some sites (urban background and urban traffic sites).

The lower biological effect observed in some sites during lockdowns with respect to the same periods in the previous year could be due to less pollutant concentrations in PM that were caused by a change in sources of air pollutant emissions (e.g., lower emissions from industries and traffic). However, the results of the present study showed that the decrease of the biological effect induced by PM during pandemic was found only in some sites. This result can be discussed considering that during lockdown some pollutant sources, such as domestic heating and waste combustion, may have been increased. Finally, differences of meteorological conditions between the two years could have influenced the PM-induced biological effects.

Finally, the different biological effects that were found using the different assays are not unexpected considering that the test methods address different endpoints. Indeed, PM is a heterogeneous mixture of different chemicals, so it is able to induce various biological effects depending on the compounds adsorbed on it that could differ according to the sampling sites (i.e., rural, urban background, urban traffic, and incinerator sites). For this reason, it is important to evaluate different biological endpoints, using a battery of short-term assays, e.g., cytotoxicity (WST-1 assay), genotoxicity (Comet assay), mutagenicity (Ames test), and estrogenic activity (gene reporter assay).

Since biological assays were proven to be sensitive enough to detect a little improvement in air quality (in terms of biological effect), the provided data may be a suggestion to design policies. Indeed, they underlined that the monitoring of pollutant concentrations alone may not be sufficient to assess air quality; therefore, policy makers should take into account that, in addition to the measurement of PM concentrations, the biological effect induced by air pollution should be also considered. With this purpose, a battery of biological assays could be included in standard monitoring programs and the air quality could be estimated using the integration of chemical and biological data, using combined indexes. Future studies are needed in order to construct these indexes and once defined, national and international legislations should establish limit values based on them rather than only on PM concentrations.

## Conclusions

After the COVID-19 pandemic, numerous studies assessed the variation of air pollutant concentrations due to containment measures but, to our knowledge, the present work is the first one in which the impact of lockdown on PM biological effects is assessed.

Despite the containment measures reducing or modifying pollutant emissions (e.g., less vehicular traffic, less industrial/commercial emissions), the result of the present study showed that these measures were not enough to reduce significantly pollutant concentrations (PM_10_, PM_2.5_, and PAHs).

Contrary to pollutant concentrations, the results of the present study showed a decrease of some biological effects of PM during the pandemic (mainly decrease of cytotoxic and genotoxic effect) which was probably due to an overall lower toxicity of the complex mixture of chemicals adsorbed on PM. Therefore, this study showed that the implementation of COVID-19 containment measures induced an improvement in air quality (assessed using biological assays).

This study also confirmed that PM biological effects cannot be assessed considering only the PM concentration especially in critical areas (such as the Padana Plain), where the geographical/meteorological conditions do not favor the dispersion of pollutants, leading to a lack of change in PM concentration despite a difference in PM emission sources. In these areas, in addition to the measurement of PM concentrations, policy makers should also consider to measure the biological effect induced by air pollution using an in vitro assay in order to protect human health from air pollution effects.

## Supplementary Information

Below is the link to the electronic supplementary material.

Supplementary file1 (DOCX 936 KB)

## Data Availability

The datasets generated during and/or analyzed during the current study are available from the corresponding author on reasonable request.
