# Supplementary material for: Biological effects of particulate matter samples during the COVID-19 pandemic: a comparison with the pre-lockdown period in Northwest Italy
Source: Air Qual Atmos Health. 2023 Jun 6:1–16. Online ahead of print. doi: 10.1007/s11869-023-01381-6 (PMC10243887; doi:10.1007/s11869-023-01381-6)
Supplement: Supplementary file 1 — Supplementary file1 (DOCX 936 KB) [file 11869_2023_1381_MOESM1_ESM.docx]

**SUPPLEMENTARY MATERIALS**

**Biological effects of particulate matter samples during the COVID-19 pandemic: a comparison with the pre-lockdown period in North-West Italy**

Marta Gea^1,#^, Manuela Macrì^2,#^, Daniele Marangon^3^, Francesco Antonio Pitasi^3^, Marco Fontana^3^, Tiziana Schilirò^1,^*, Sara Bonetta^1^

^1^Department of Public Health and Pediatrics, University of Torino, via Santena 5 bis, 10126 Torino, Italy

^2^Department of Life Sciences and Systems Biology, University of Torino, via Accademia Albertina 13, 10123 Torino, Italy

^3^Regional Agency for Environmental Protection of Piedmont (ARPA Piemonte), via Sabaudia 164, 10095 Grugliasco, Italy

#Marta Gea and Manuela Macrì equally contributed to this work

*Corresponding author:

Tiziana Schilirò

tiziana.schiliro@unito.it;

Department of Public Health and Pediatrics,

University of Torino,

Via Santena 5 bis, 10126 Torino, Italy.

Phone: +39 0116705820

*Air Quality, Atmosphere & Health*

**Fig. S1** Cytotoxicity of 2019 PM extracts at 24 h and 72 h in the rural site: (a) January/February PM extracts, (b) March PM extracts; (c) April PM extracts; (d) May/June PM extracts; (e) Summer PM extracts; (f) Autumn PM extracts. Data are expressed as means ± standard deviations.


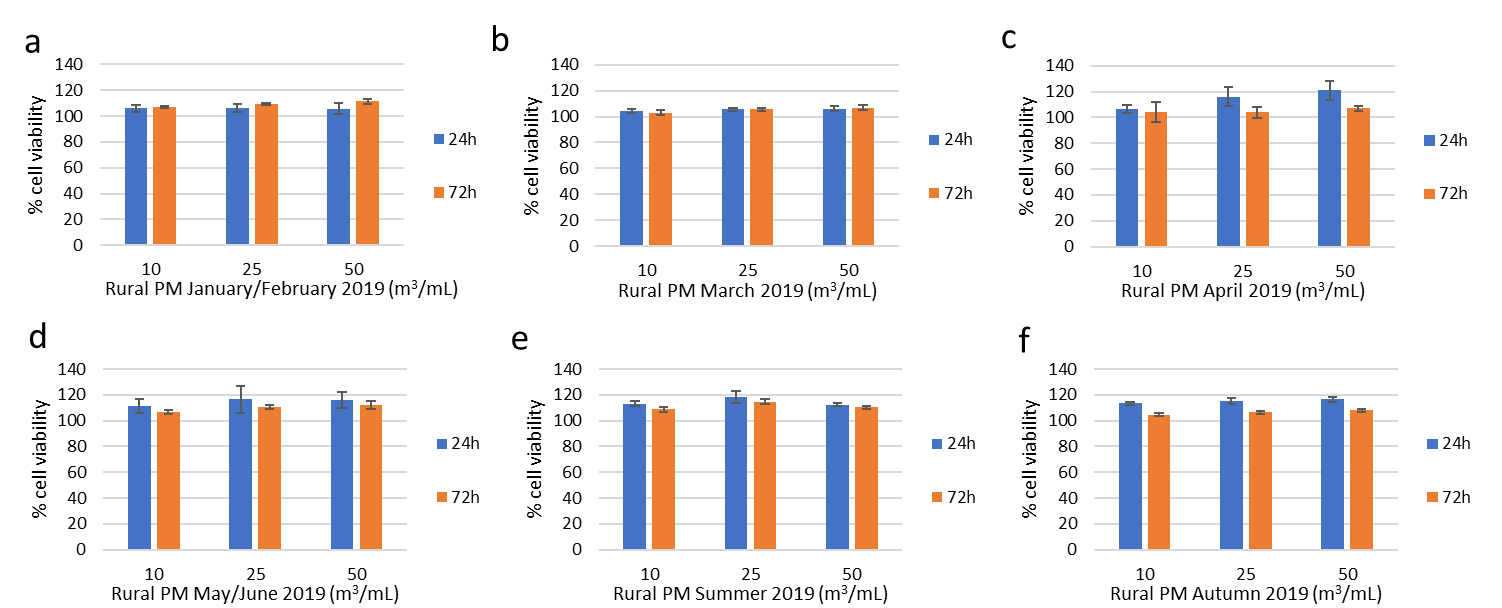


**Fig. S2** Cytotoxicity of 2020 PM extracts at 24 h and 72 h in the rural site: (a) January/February PM extracts, (b) March PM extracts; (c) April PM extracts; (d) May/June PM extracts; (e) Summer PM extracts; (f) Autumn PM extracts. Data are expressed as means ± standard deviations.


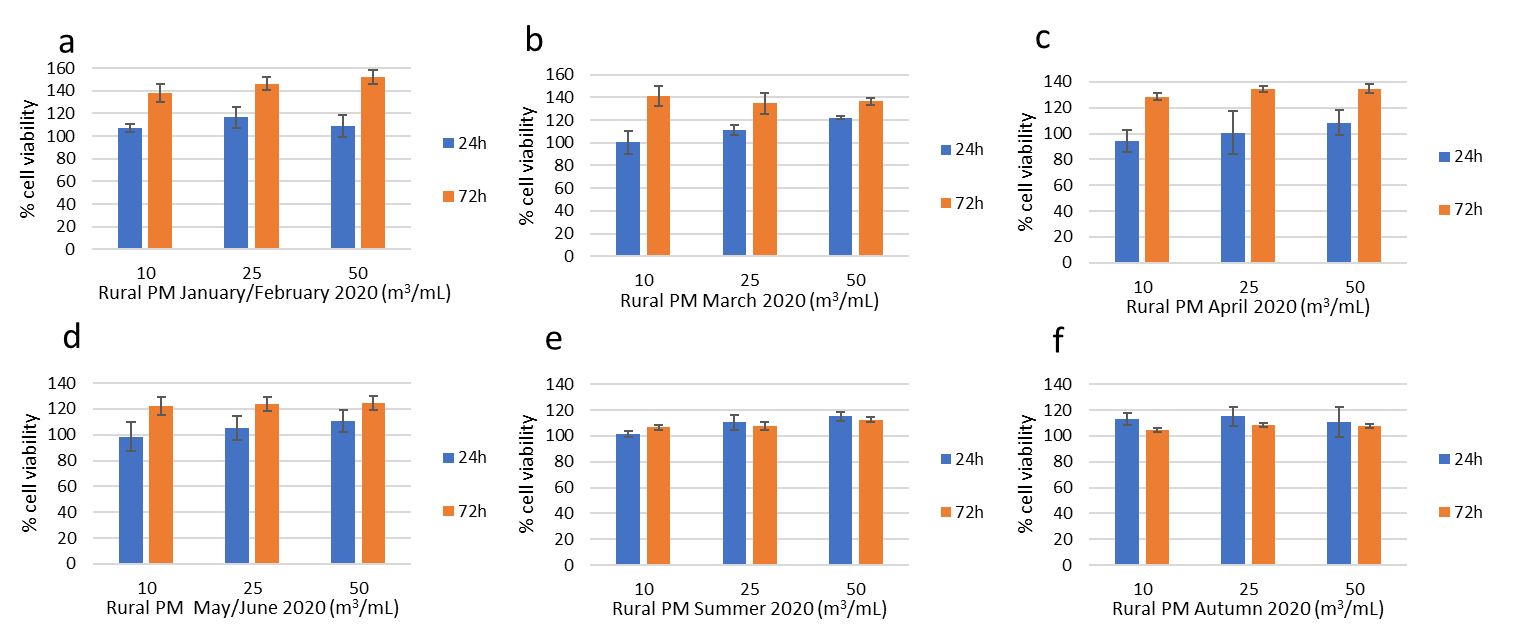


**Fig. S3** Cytotoxicity of 2019 and 2020 PM extracts at 24 h and 72 h in the urban background, urban traffic and incinerator sites during April, May/June and Summer. (a) Urban PM April 2019, (b) Traffic PM April 2019; (c) Incinerator PM April 2019; (d) Urban PM May/June 2019; (e) Traffic PM May/June 2019; (f) Incinerator PM May/June 2019; (g) Urban PM Summer 2019; (h) Traffic PM Summer 2019; (i) Incinerator PM Summer 2019; (j) Urban PM April 2020, (k) Traffic PM April 2020; (l) Incinerator PM April 2020; (m) Urban PM May/June 2020; (n) Traffic PM May/June 2020; (o) Incinerator PM May/June 2020; (p) Urban PM Summer 2020; (q) Traffic PM Summer 2020; (r) Incinerator PM Summer 2020. Data are expressed as means ± standard deviations.


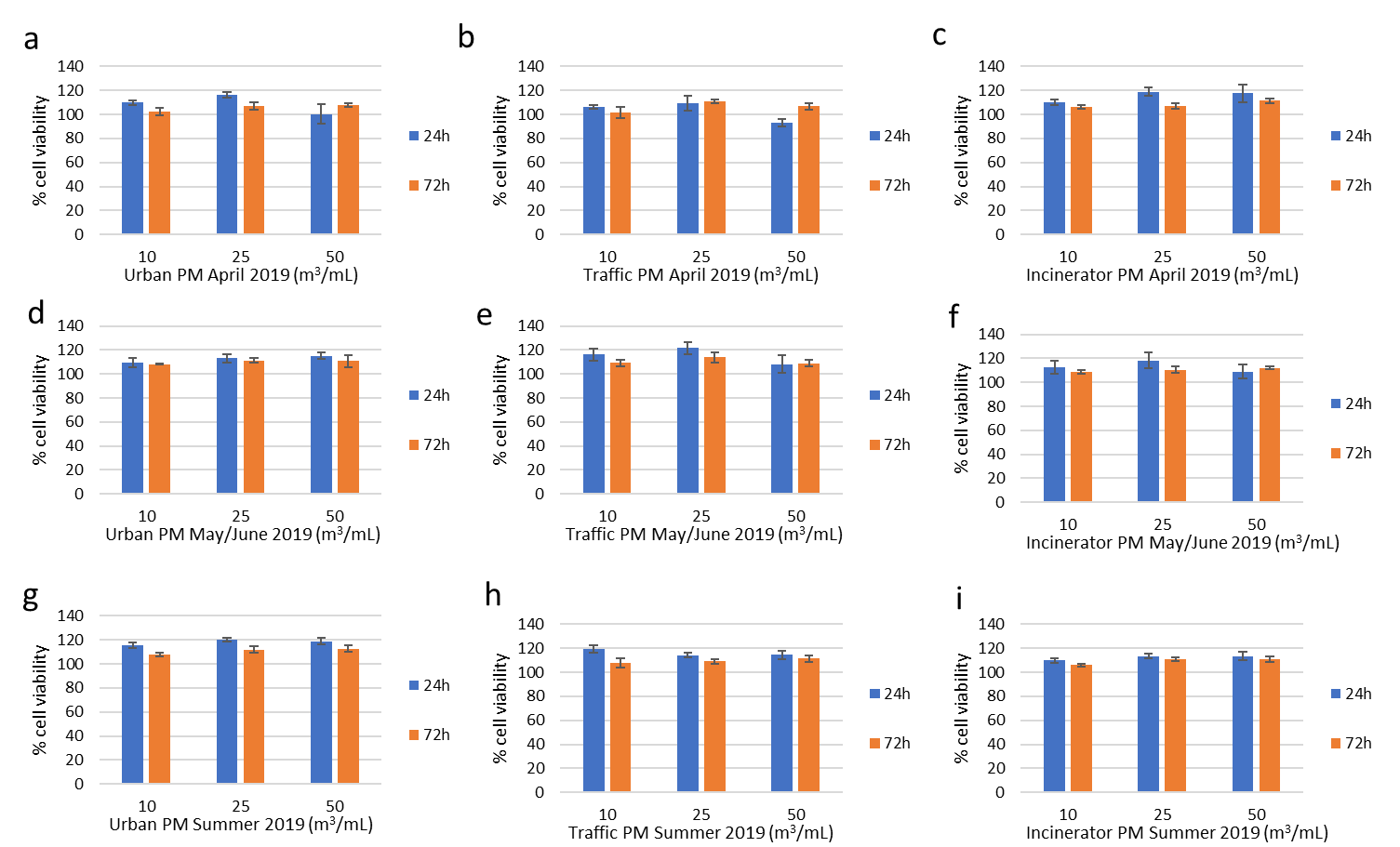


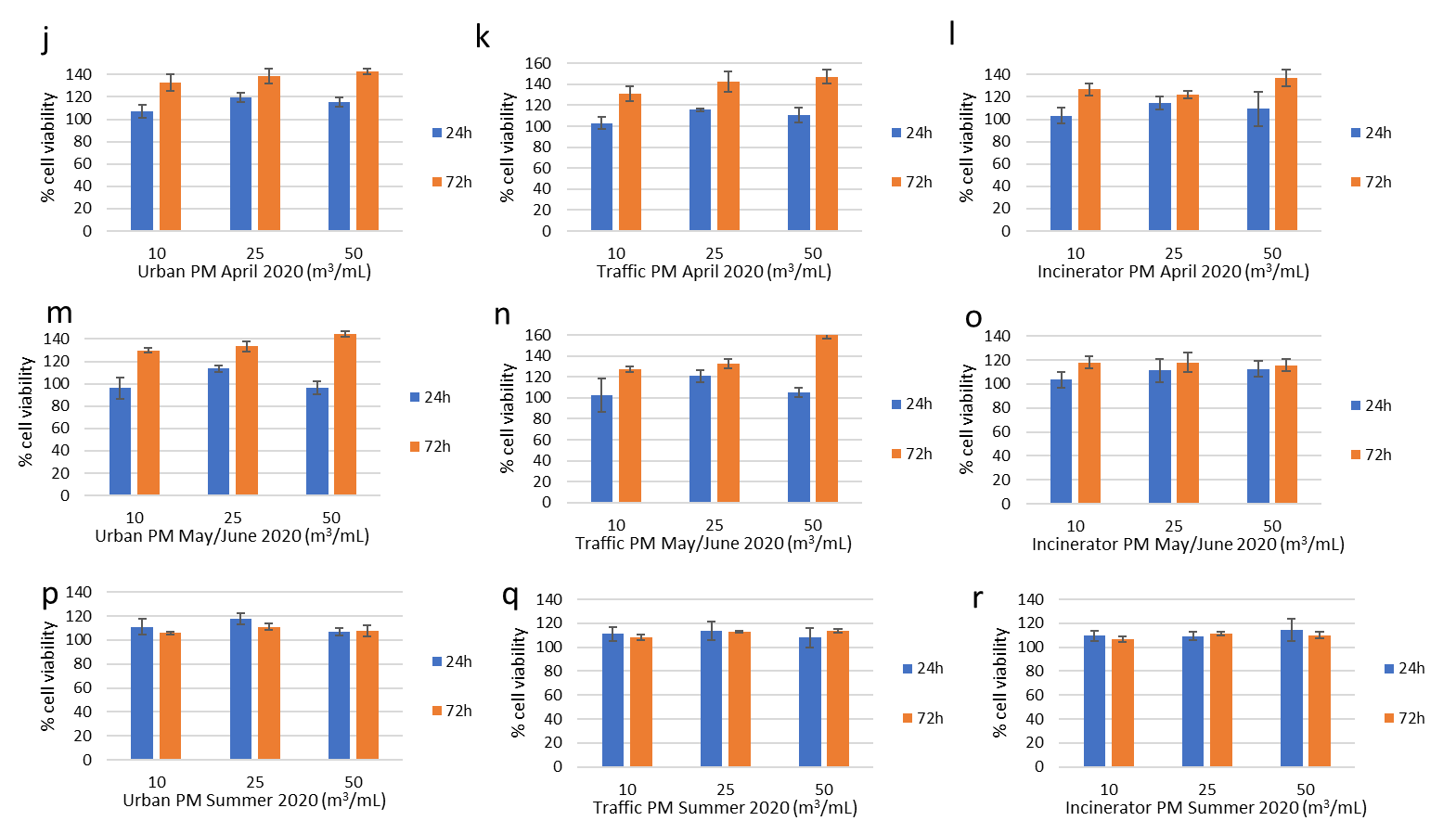


**Fig. S4** Cytotoxicity of PM extracts at 72 h: (a) January/February PM extracts (no restrictions), (b) March PM extracts (first lockdown, hard restrictions); (c) Autumn PM extracts (second lockdown, intermediate restrictions). PM rural extracts (2019 and 2020, 24 h and 72 h) did not induce any cytotoxic effect (data not shown). Data are expressed as means ± standard deviations.*p≤ 0.05 Kruskal-Wallis test followed by Dunnett’s post hoc test vs negative control


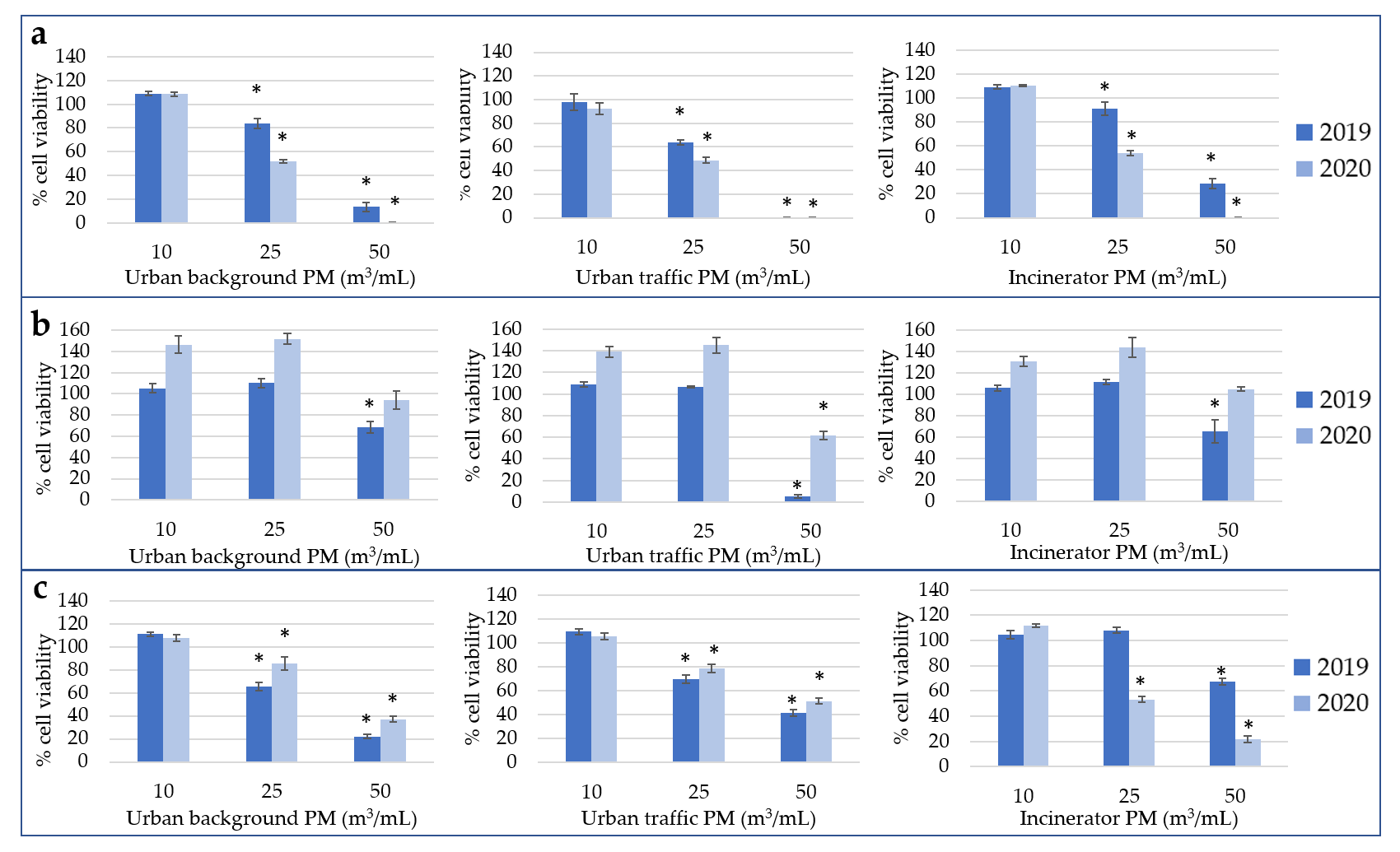


**Fig. S5** Genotoxicity of May/June PM extracts at 24 h exposure: (a) urban background PM, (b) urban traffic PM, (c) rural PM, (d) incinerator PM. Data are expressed as means ± standard deviations.*p≤0.05 one-way ANOVA test followed by Dunnett’s post hoc test *vs*. negative control

**
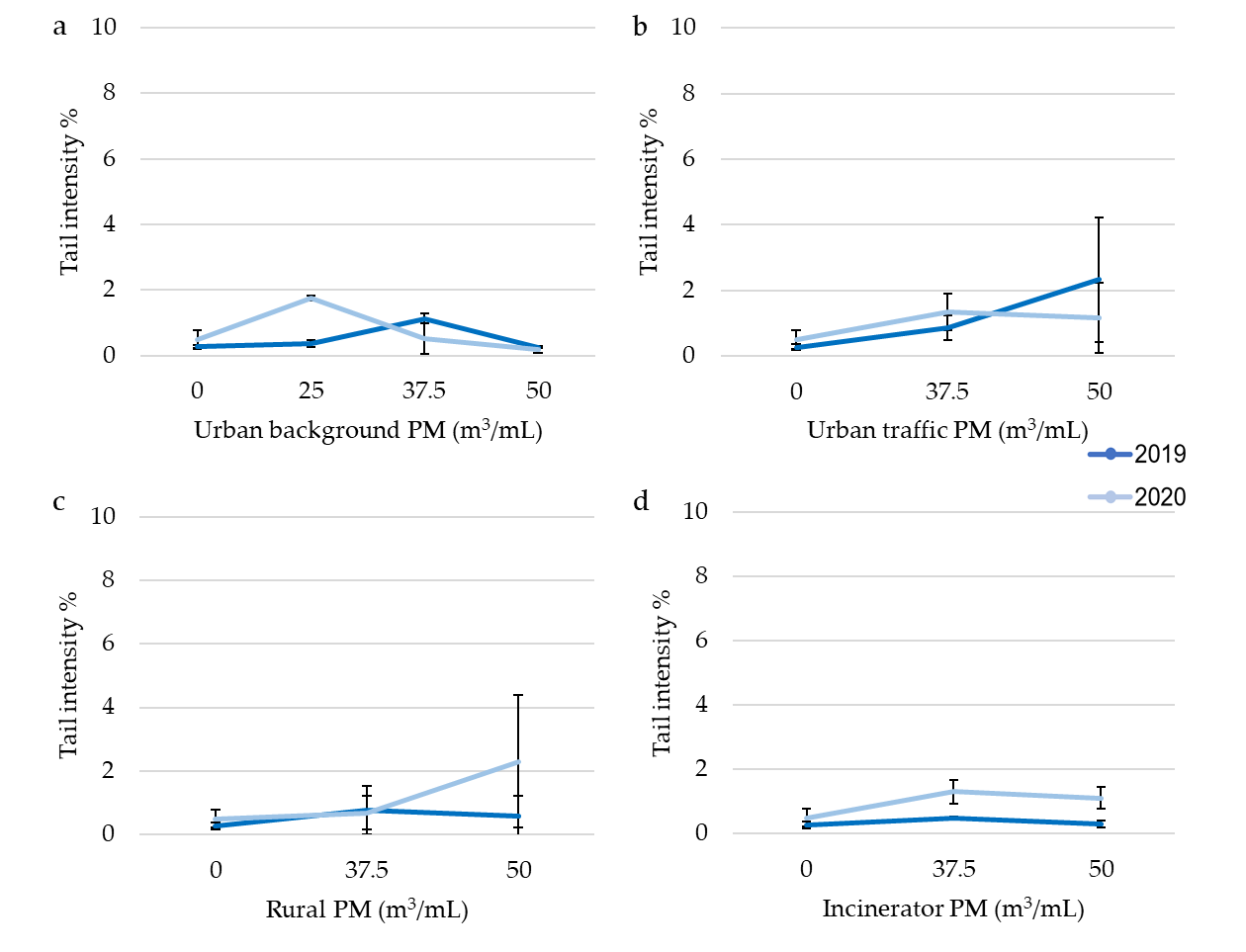
**

**Fig. S6** Genotoxicity of Summer PM extracts at 24 h exposure: (a) urban background PM, (b) urban traffic PM, (c) rural PM, (d) incinerator PM. Data are expressed as means ± standard deviations.*p≤0.05 one-way ANOVA test followed by Dunnett’s post hoc test *vs*. negative control

**
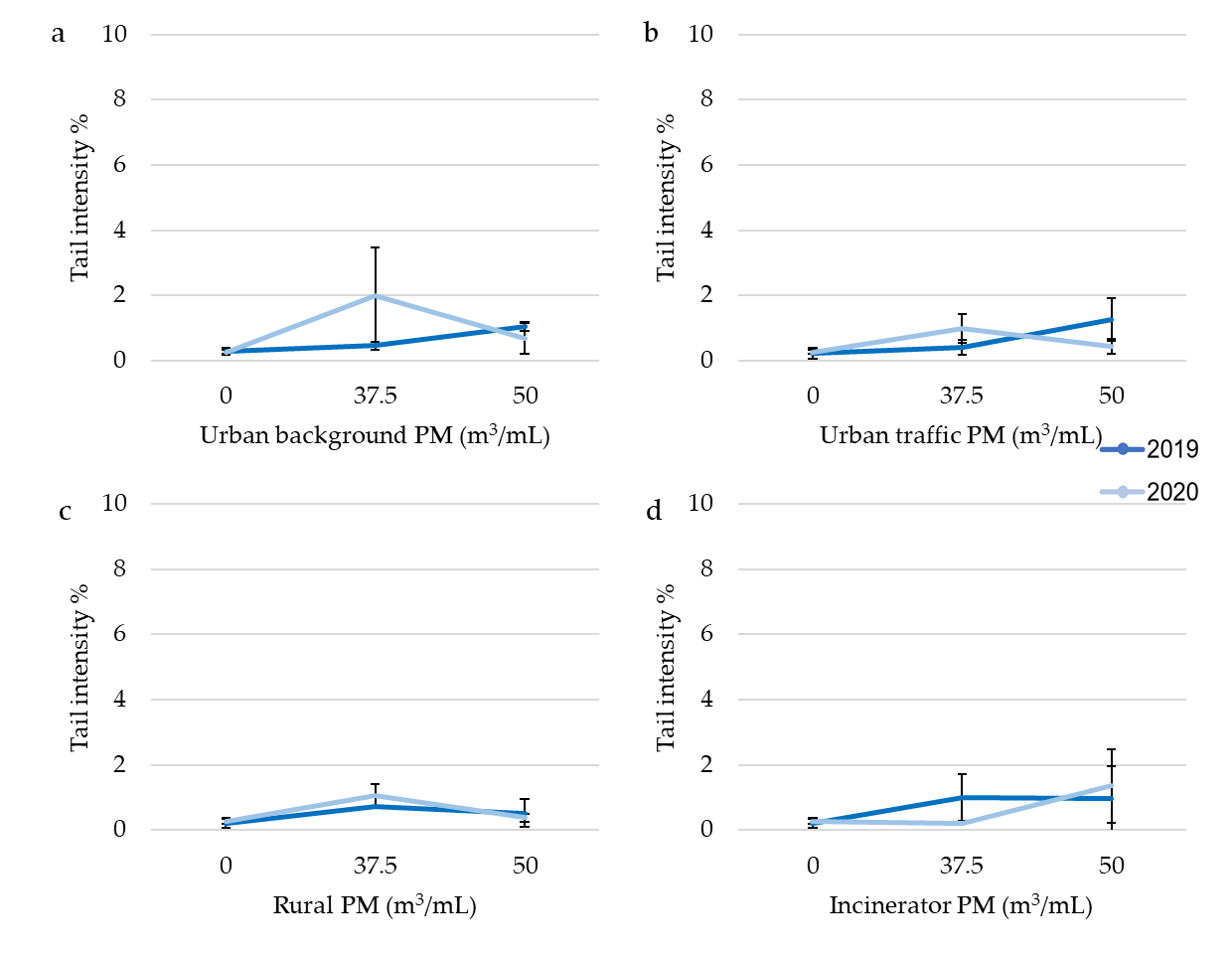
**

**Fig. S7** Genotoxicity of January/February PM extracts at 24 h exposure: (a) urban background PM, (b) urban traffic PM, (c) rural PM, (d) incinerator PM. Data are expressed as means ± standard deviations.*p≤0.05 one-way ANOVA test followed by Dunnett’s post hoc test *vs*. negative control

**
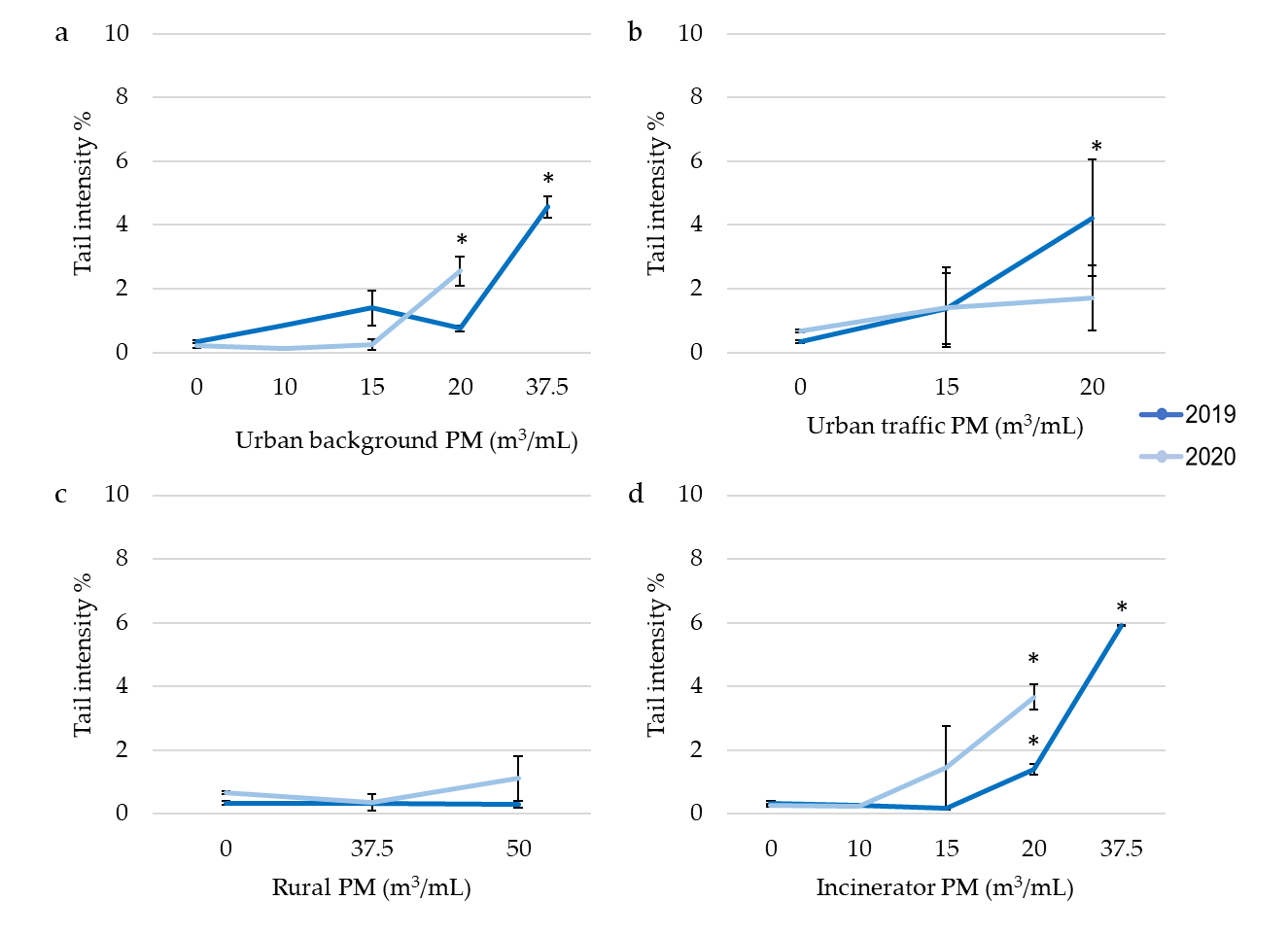
**

**Fig. S8** Genotoxicity of March PM extracts at 24 h exposure: (a) urban background PM, (b) urban traffic PM, (c) rural PM, (d) incinerator PM. Data are expressed as means ± standard deviations.*p≤0.05 one-way ANOVA test followed by Dunnett’s post hoc test *vs*. negative control

**
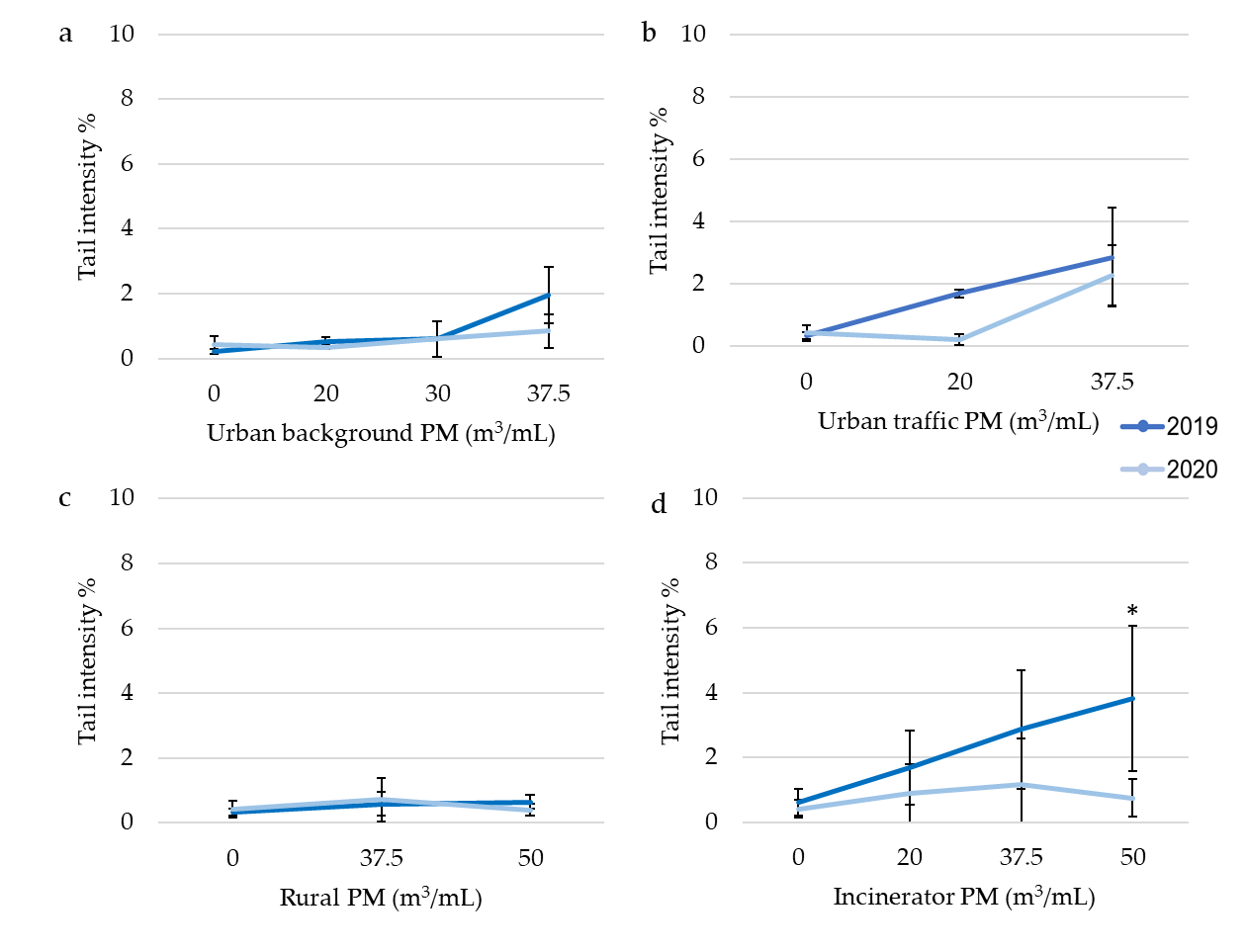
**

**Fig. S9** Genotoxicity of April PM extracts at 24 h exposure: (a) urban background PM, (b) urban traffic PM, (c) rural PM, (d) incinerator PM. Data are expressed as means ± standard deviations.*p≤0.05 one-way ANOVA test followed by Dunnett’s post hoc test *vs*. negative control

**
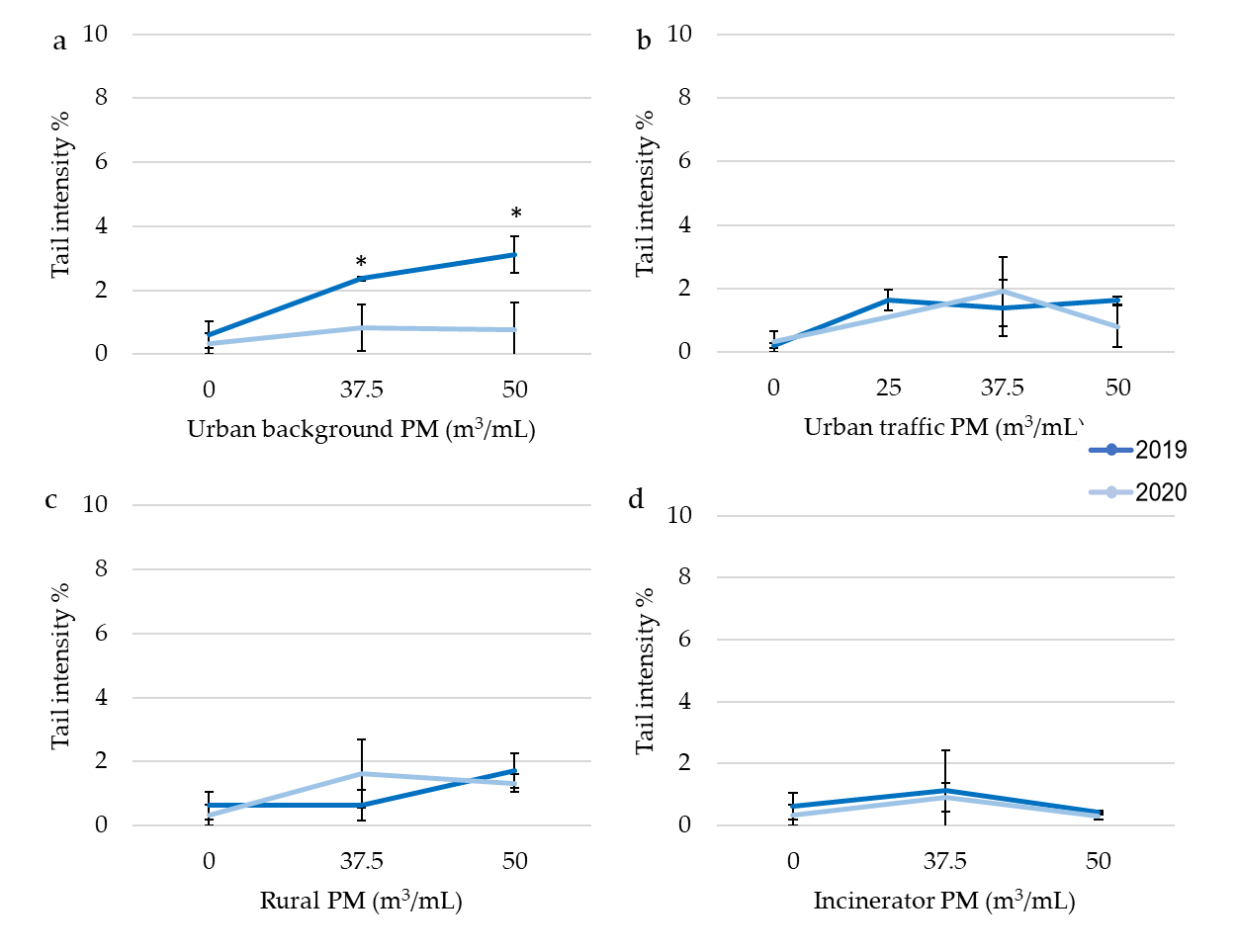
**

**Table S1** Number of strains on which the extracts induced a significant mutagenic effect (MR ≥1). Strains: TA98, TA98 + S9, TA100, TA100 + S9

| Period | Urban background site | | Urban traffic site | | Rural site | | Incinerator site | |
| --- | --- | --- | --- | --- | --- | --- | --- | --- |
|  | **2019** | **2020** | **2019** | **2020** | **2019** | **2020** | **2019** | **2020** |
| January/  February | 4 | 4 | 4 | 4 | 2 | 1 | 4 | 4 |
| March | 3 | 2 | 4 | 3 | 1 | 0 | 4 | 3 |
| April | 2 | 0 | 1 | 2 | 0 | 0 | 0 | 2 |
| May/June | 0 | 0 | 0 | 0 | 0 | 0 | 0 | 0 |
| Summer (July-August, September) | 0 | 0 | 0 | 0 | 0 | 0 | 0 | 0 |
| Autumn (October, November, December) | 3 | 4 | 4 | 4 | 1 | 2 | 4 | 4 |

|  |
| --- |
